# Reliability of Total Grain-Size Distribution of Tephra Deposits

**DOI:** 10.1038/s41598-019-46125-8

**Published:** 2019-07-10

**Authors:** L. Pioli, C. Bonadonna, M. Pistolesi

**Affiliations:** 1 Dipartimento di Scienze Chimiche e Geologiche, Università di Cagliari, Cagliari, Italy; 2 Département des Sciences de la Terre, Université de Genève, Genève, Switzerland; 3 Dipartimento di Scienze della Terra, Università di Pisa, Pisa, Italy

**Keywords:** Natural hazards, Volcanology

## Abstract

Total Grain-Size Distribution (TGSD) of tephra deposits is key to the characterization of explosive volcanism, plume-dispersal modeling, and magmatic fragmentation studies. Nonetheless, various aspects that includes deposit exposure and data fitting make its determination extremely complex and affect its representativeness. In order to shed some lights on the reliability of derived TGSDs, we examine a large TGSD dataset in combination with a sensitivity analysis of sampling strategies. These analyses are based both on a well-studied tephra deposit and on synthetic deposits associated with a variety of initial eruptive and atmospheric conditions. Results demonstrate that TGSDs can be satisfactorily fitted by four distributions (lognormal, Rosin-Rammler, and power-law based either on the absolute or cumulative number of particles) that capture different distribution features. In particular, the Rosin-Rammler distribution best reproduces both the median and the tails of the TGSDs. The accuracy of reconstructed TGSDs is strongly controlled by the number and distribution of the sampling points. We conclude that TGSDs should be critically assessed based on dedicated sampling strategies and should be fitted by one of the mentioned theoretical distributions depending on the specific study objective (e.g., tephra-deposit characterization, physical description of explosive eruptions, tephra-dispersal modeling).

## Introduction

The grain-size distribution of the eruptive mixture injected into the atmosphere during volcanic explosive eruptions is an important property of eruptive dynamics and represents a critical parameter both for magma fragmentation studies and for the description of tephra transport and sedimentation^1–5^. It is also one of the most difficult to constrain out of all eruption source parameters (ESPs) needed for numerical simulations of plume and cloud dispersal (e.g., total grain-size distribution, plume height, mass eruption rate, erupted mass^6^).

The grain-size of the eruptive mixture associated with explosive eruptions is primarily controlled by magma fragmentation, which is driven by multiple simultaneous processes depending on magma properties (e.g., viscosity, porosity, and permeability), and flow-controlled parameters, such as shearing and gas expansion rates^7,8^. Measurements of the size of settling particles in real time represent the ideal technique for grain-size distribution assessment, because they are not affected by issues associated with deposit erosion, remobilization, and contamination with previous or later eruptive phases, which can strongly affect deposit-based strategies^9^. However, due to various technical limitations, no instrument (e.g., satellite sensors, radars, disdrometers) can currently provide comprehensive information on the total size range of particles dispersed in the atmosphere and settling on the ground^10^. For this reason, forecasting of tephra dispersal is mostly performed considering specific meteorological conditions and column height, but grain-sizes are based on known tephra-deposit characteristics, either generically assumed^3,11^ or calculated through statistical models^12^.

For simplicity, in this paper with Total Grain-Size Distribution (TGSD) we refer to the grain-size distribution of the whole eruptive mixture ejected during an explosive eruption. It can be recorded as the best available approximation by the size distribution of tephra-fallout deposits emplaced on the ground. It is important to note that the size distribution at individual tephra outcrops (i.e., of the mixture of particles falling at any given distance from the vent) is not representative of the TGSD^4,13,14^. For this reason, TGSDs needs to be interpolated from grainsize distribution of tephra at different locations^12,15–18^. The accuracy of determination of TGSDs, depends not only on the representativeness of the size distribution of the samples collected at each location, which in turn relies on sampling methods and sampled volume, but also both on the number of measuring stations/outcrops and their spatial distribution^6,15^. TGSDs are typically calculated based on the weighted average of distributions of individual locations either taking into account the deposit mass load per unit area at each sample location or using the Voronoi tessellation strategy^15^. This second method accounts both for the deposit mass load and for the size distribution of particles at each sampling location. In fact, the tephra deposit is divided into Voronoi cells whose interior consists of all grid points which are closer to a particular sample point than to any other. Then the TGSD is obtained as the area-weighted average of all the Voronoi cells over the whole deposit.

Traditionally, distributions are studied in terms of empirical parameters including median size and standard deviation^19^, with very few attempts to define formal distributions based on comparative studies on known TGSDs or theoretical studies based on fragmentation theories^1,12,20,21^.

Based on a large dataset of most published TGSDs up to date (Table 1), we present a comparative study to investigate their average properties and define the best fitting distribution models; we then analyze and discuss the results in terms of representativeness and stability of the distribution taking into account sampling issues due to partial preservation or poor exposure of the deposits. The fitting strategies are finally tested on synthetic deposits which combine a range of ESPs and atmospheric parameters (e.g., known TGSD, column height, and wind speed) with variable geometries of sampling sites. Results are used to provide general criteria for the determination and numerical description of TGSDs as reliable proxies for the initial grain-size distribution of volcanic particles injected into the atmosphere. Because no or very few details (i.e., the volume of material, the numbers of layers sampled) is usually provided on the specific sampling techniques adopted in each deposit of the dataset, we assume that provided GSDs are in all cases representative of the GSDs of the location where they were collected, and discard any possible uncertainty deriving from partial, or insufficient sampling of the tephra deposit at any single location. For this reason, the representativeness of the TGSDs dataset will be discussed only in terms of number and distribution of outcrops.

**Table 1:** Eruptions considered in this study, magma composition, column height, Mass Eruption Rate (MER), method of TGSD calculation (V = Voronoi, W A = Weighted Average on mass load, I W A = Isopach-based Weighted Average) and reference details.

Eruption	Bulk rock composition	Column height above vent (km)	MER (kg/s)	Calculation method	Main reference
Etna 19-24/07/2001	Basalt	2.5	7.4E + 03	V	^44^
Etna 27/10/2002	Basalt	3.25	3.0E + 05	V	^45^
Etna 24/11/2006	Basalt	0.8	5.0E + 03	V	^46^
Etna 4-5/09/2007	Basalt	2	5.0E + 03	V	^47^
Etna 12-13/01/2011	Basalt	7	2.5E + 04	V	^48^
Etna 18-19/05/2016	Basalt	2–2.5	2.0E + 3	V	^49^
Etna 21/05/2016	Basalt	2–3	7.1E + 02	V	^49^
Izu Oshima 1986	Basaltic andesite	12	1.1E + 05	W A	^50^
Fuego 1974	Basaltic andesite	10	3.0E + 06	V	^51^
Heimaey 1973	Basalt	—	1.0E + 05	W A	^52^
Hekla 1845	Basaltic andesite	19	2.1E + 07	V	^53^
Hekla 1991	Basalt	11	3.4E + 06	V	^54^
Hekla 2000	Basaltic andesite	11	7.2E + 07	V	^41^
Hekla 1104	Rhyolite	20–25	1.2 E + 08	V	^55^
Hekla 1300-D	Dacite	25	1.2 E + 08	V	^55^
Hekla 1693	Andesite	18	3.2E + 07	V	^55^
Hekla 1766	Andesite	18	3.2E + 07	V	^55^
Tecolote	Basalt	11	3.2E + 6	V	^56^
Kilauea Iki, 1959	Basalt	0.6	6.3E + 05	W A	^57^
Eyjafjallajokull 4-8/05/2010	Benmoreite-Trachyte	7	8.0E + 04	V	^10^
St. Vincent 1979	Basaltic andesite	11	6.0E + 06	W A	^58^
Chaiten 06/05/2011	Rhyolite	19	—	V	^59^
Al Madinah 1256	Basalt	—	—	W A	^60^
Ruapehu 1996	Andesite	6	1.5E + 05	V	^15^
Mt Spurr Aug 1992	Andesite	12	1.7E + 06	W A	^61^
Mt Spurr Sept 1992	Andesite	12	1.8E + 06	W A	^61^
Soufriere Hills 31/03/1997	Andesite	—	—	W A	^62^
Soufriere Hills 12/09/1997	Andesite	4	—	W A	^62^
Soufriere Hills 15/09/1997	Andesite	—	—	W A	^62^
Soufriere Hills 21/09/1997	Andesite	—	—	W A	^62^
Soufriere Hills 26/09/1997	Andesite	11	3.0E + 06	W A	^62^
Soufriere Hills 28/09/1997	Andesite	—	—	W A	^62^
Soufriere Hills 01/10/1997	Andesite	—	—	W A	^62^
Soufriere Hills 02/10/1997	Andesite	—	—	W A	^62^
Soufriere Hills 10/10/1997	Andesite	—	—	W A	^62^
Soufriere Hills 18/07/2005	Andesite	10	1.0E + 06	V	^63^
Soufriere Hills 27/07/2005	Andesite	7	1.0E + 06	V	^63^
Mt. St. Helens 18/05/1980	Dacite	20	1.9E + 07	I W A	^26^
Cordón Caulle 2011 Unit I	Rhyolite	8-12	5.0E + 06	V	^16^
Askja 1875 phase C	Rhyolite	23	1.0E + 08	W A	^64^
Askja 1875 phase D	Rhyolite	26	8.2E + 07	W A	^64^
Vesuvius 1906 L2	K tephrite	12	1.0E + 06	V	^65^
Vesuvius 1906 L3	K tephrite	3-4	1.0E + 05	V	^65^
Vesuvius 1906 ash	K tephrite	6-7	—	V	^65^
Baia	Trachyte	17	4.0E + 10	V	^66^
Pululagua 2450 BP	Dacite	25 ± 5	—	V	^18^
El Chichon 1982	Trachyandesite	27	4.0E + 07	W A	^67^
Cotopaxi layer 3	Andesite	23	4.8E + 08	V	^68^
Cotopaxi layer 5	Basaltic andesite	26	6.0E + 07	V	^68^
Rungwe pumice	Trachyte	30–35	2-5E + 08	V	^69^

## Results

### General significance of tested statistical distributions

Below we show the results of the fitting of empirical data (i.e., TGSD derived from field data) according to the lognormal and the Rosin-Rammler (equivalent to the Weibull distribution^22^), based on the weight distribution, and the power-law distributions, based either on the absolute or cumulative number of particles. Despite their ability in fitting the TGSDs, each of these distributions has different potential and limitations due to their theoretical basis and constitutive assumptions. While lognormal distributions are fitted based on (and thus better reproducing) the median and standard deviation of the distribution, the Rosin-Rammler distributions, being cumulative, better describe its tails. Power-law distributions are, in contrast, not suitable to describe the tails of the distribution.

**Figure 1:**
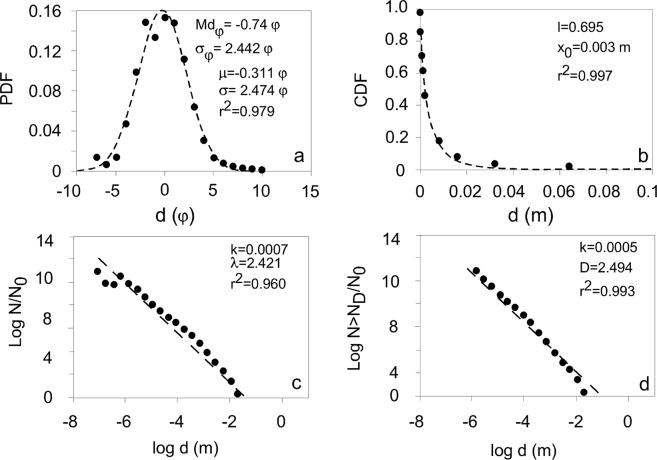
Examples of distribution fittings of the 1996 Ruapehu eruption TGSD. (**a**) Lognormal (Eq. 8); (**b**) Rosin-Rammler (Eq. 7); (**c**) Power-law (Eq. 4); (**d**) Cumulative power-law (Eq. 5). Dashed lines describe the fitted distributions. CDF = cumulative density function, PDF = probability density function, d = particle diameter, N/N_0_ = normalized number of particles, N > N_D_/N_0_ = normalized number of particles with diameter larger than (**d**). The main parameters of each distribution fitting are enclosed in each graph.

As an example, the shapes of the four main distribution types fitted to the TGSD of the 1996 Ruapehu (New Zealand) are shown in Fig. 1.

#### Weight vs. particle size distributions (lognormal, Rosin-Rammler)

**Figure 2:**
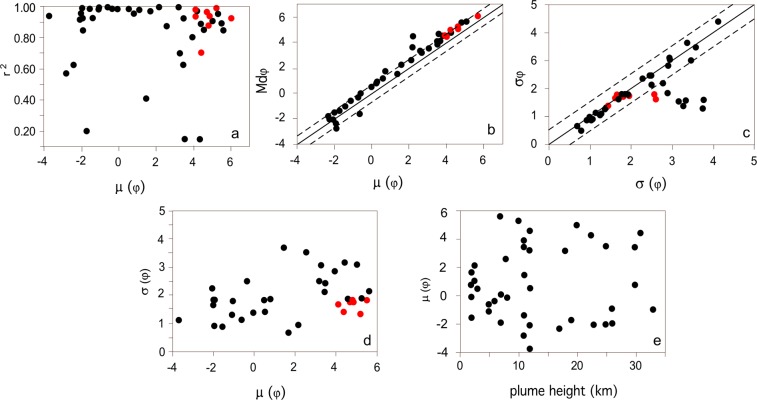
Results of lognormal fitting of the TGSDs. (**a**) Pearson correlation coefficient r^2^ of the lognormal fitting vs. median grain-size of the distribution (μ); (**b**) median of the empirical distribution (Md_ϕ_) vs. median grain-size of the distribution (μ); (**c**) sorting (σ_ϕ_) of the empirical distribution vs. standard deviation of the lognormal distribution (σ); (**d**) μ vs. σ; (**e**) μ vs. plume height of the associated eruptions. Red circles: size distributions of co-PDC deposits. Black circles: all other size distributions.

The lognormal distribution satisfactorily fits TGSDs for only about 60% of the dataset, with a Pearson correlation coefficient r^2^ > 0.9 (Fig. 2a). Despite this, the fitted median grain-size and standard deviation are within half ϕ (difference from median (Md_ϕ_) and sorting (σ_ϕ_) of the empirical distribution (Fig. 2b,c) in most cases, with ϕ being −log_2_d and d the particle diameter in millimeters. There is no apparent correlation between (fitting) median μ and standard deviation σ (Eq. 7), both showing a large variability (Fig. 2d; Table 1 in Supplementary Material). Finally, as already observed by previous studies^12^, no clear relationship between σ and eruptive column height can be defined (Fig. 2e).

**Figure 3:**
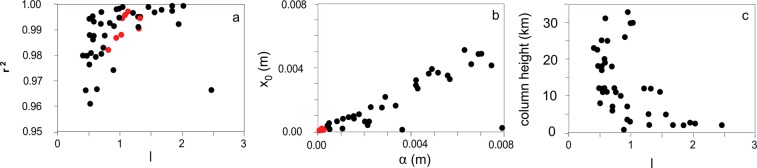
(**a**) Pearson correlation coefficient r^2^ of the Rosin-Rammler distribution fitting vs. *l* of the studied distributions. (**b**) Variability of the parameter *x*_0_ of the Rosin-Rammler distribution fitting vs. the empirical median diameter (α) of the studied distributions. The dashed line indicates the best-fit linear correlation between the two parameters. (**c**) Variability of the parameter *l* vs. column height of the associated eruptions. Symbols as in Fig. 2.

The Rosin-Rammler distribution fits very well all the datasets, with the Pearson correlation coefficient always being >0.94 (Fig. 3a). In literature, the length scale parameter *x*_0_ (Eq. 8) has been empirically linked with various percentiles of the original distribution^22^. For the dataset studied here, it correlates linearly with the median size *α* of the empirical distribution, expressed in the same linear unit (m), with the relationship (Fig. 3b):

1 $${x}_{0}=\frac{1}{3}\alpha $$

with r^2^ = 0.9996

The shape parameter *l* (Eq. 8) quantifies both the material properties and the fragmentation process. In our results, it varies from 0.5 to 2.5, and is <1.5 in ~85% of the studied eruptions. Smaller eruptions (with column heights <10 km) display the largest variability in terms of *l*, varying between 2.5 and 0.9, whereas larger eruptions have lower *l* ranging from 1 to 0.5 (Fig. 3c).

#### Particle number vs. particle size distributions (power-law)

**Figure 4:**
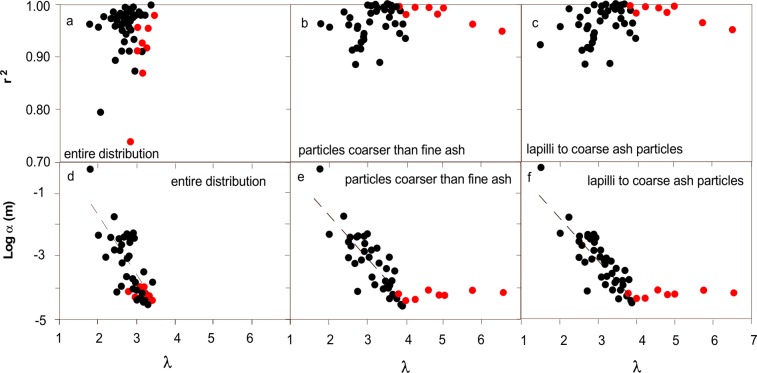
Variability of *λ* versus Pearson correlation coefficient r^2^ for number of particles obtained from the power-law fitting of all the TGSDs considered (Eq. 2, Table 1); (**a**) entire distribution; (**b**) particles coarser than fine ash (>0.063 mm); (**c**) lapilli (64–2 mm) to coarse ash (2–0.063 mm) particles. Variability of median particle diameter α versus *λ*: (**d**) calculated over the entire distribution; (**e**) calculated only for distribution comprising particles coarser than fine ash; (**f**) lapilli to coarse ash. Dashed lines are best fitting lines calculated for the empirical dataset not taking into account co-PDC distributions. Symbols as in Fig. 2.

A simple power-law fitting of the number of particles vs. their size gives the best results, with r^2^ > 0.9 in more than 90% of the distributions (Fig. 4a) and the lowest value of 0.74 corresponding to a co-PDC deposit. Generally, a deviation from the typical power-law trend is seen at the smallest particle size classes, and in some cases also at the largest ones (Fig. 1c), marking the size range of validity of the distribution (see discussion in Section 3).

**Figure 5:**
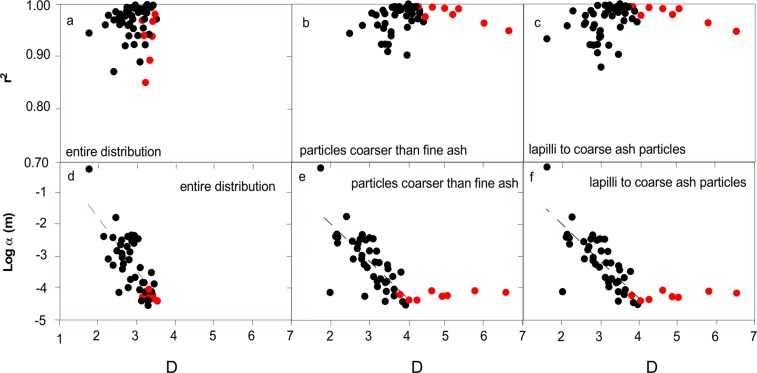
Variability of D versus Pearson correlation coefficient r^2^ obtained from the power-law fitting of cumulative number of particles all the TGSDs considered (Eq. 5, Table 1); (**a**) entire distribution; (**b**) particles coarser than fine ash (<0.64 mm); (**c**) lapilli (64–2 mm) to coarse ash (2–0.063 mm) particles. Variability of median particle diameter α versus: (**d**) D calculated over the entire distribution. (**e**) D calculated only for particles coarser than fine ash; and (**f**) lapilli to coarse ash. Symbols as in Fig. 2. Dashed lines are best fitting lines calculated for the empirical dataset not taking into account co-PDC distributions (red dots).

For the reasons above, the fitting improves when considering only particles coarser than fine ash (≤5 ϕ; Fig. 4b) or particles with size comprised from lapilli (<−6 ϕ) to coarse ash (<−1 ϕ) (Fig. 4c). The slopes of the distributions *λ* range from 1.8 to 3.5 when considering all the particle sizes and increases up to 6.5 in reduced size range distributions (Fig. 4d–f), with the larger values attained by co-PDC particle distributions. Cumulative particle number (Eq. 5) shows even better fitting, with r^2^ consistently >0.85 when fitting the complete size range (Fig. 5a) and >0.91 when fitting only the distribution of particles larger than fine ash or lapilli to coarse ash (Fig. 5b,c), respectively. The slopes of the distributions show very similar variability with respect to simple power-law curves. In both cases (power-law and cumulative power-law fittings), the slopes increase with increasing median particle diameter α of the distribution (Fig. 5d–f). The weak correlation between slope *D* and the median size α (expressed in mm scale) becomes significant when excluding the tail of the distributions (i.e., when considering only coarse ash and coarse ash and lapilli), with the exception of co-PDC deposits. Co-PDC deposits show larger deviations from the main trend when excluding fine particles from the computations (Figs 4d–f and 5d–f).

The correlations shown in Figs 4 and 5 can be expressed as:

2 $$\mathrm{log}\,{\rm{\alpha }}=a{\rm{\lambda }}+{\rm{b}}$$

3 $$\mathrm{log}\,{\rm{\alpha }}={{\rm{a}}}_{{\rm{1}}}{\rm{D}}+{{\rm{b}}}_{{\rm{1}}}$$

When α is expressed in m, the fitting parameters as calculated from the dataset are:

a = −1.91, b = 2.12 with r^2^ = 0.50 and a_1_ = −1.71, b_1_ = 1.68 with r^2^ = 0.50 calculated over the entire distribution; a = −1.38, b = 1.16 with r^2^ = 0.65, a_1_ = −1.48, b_1_ = 1.52 with r^2^ = 0.68 calculated excluding the fine ash component; and a = −1.42, b = 1.26 with r^2^ = 0.72 a_1_ = −1.48, b_1_ = 1.49 with r^2^ = 0.72 calculated for the distribution of lapilli to coarse ash particles.

### Stability of computed grain-size distribution

#### Sensitivity analysis based on a well-studied tephra deposit

**Figure 6:**
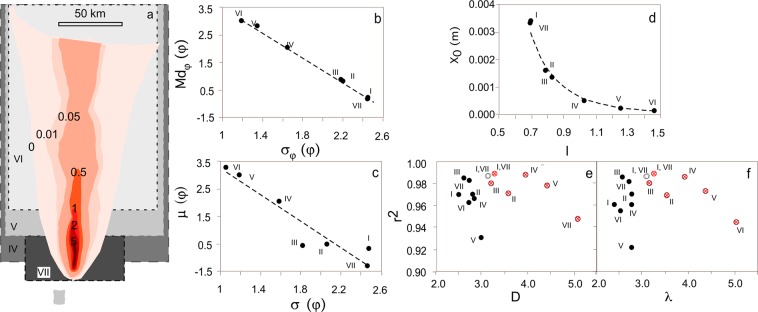
Variability of fitting parameter for TGSD estimated for the Ruapehu 1996 eruption tephra blanket using different sites combinations (see main text for more details). (**a**) Map of the deposit of the Ruapehu 1996 eruption (areas shaded in red tones). Different red tones areas mark isolines of mass distribution (numbers represent mass loads in kg/m^2^), based on^38^. The grey areas and roman numbers highlight the areas sampled for combinations IV, V, VI, and VII. (see text for details). (**b**) Md_ϕ_ and sorting of the distributions; (**c**) fitting parameters of the lognormal distribution; (**d**) *x*_0_ and *l* parameters of the Rosin-Rammler distribution. (**d**) Pearson correlation coefficient r^2^ vs. D of the fitted cumulative power-law distribution. (**e**) Pearson correlation coefficient r^2^ vs. D of the fitted cumulative power-law distribution. (**f**) Pearson correlation coefficient r^2^ vs. *λ* of the fitted power-law distribution. In plots (**e**, **f**) black dots represent distribution calculated over the entire particle size range, open black dots refer to distribution of particles coarser than fine ash, red crossed dots refer to distributions calculated over the lapilli particle sizes only. These last two types of distributions overlap in most cases. Points are labelled according to the different sampling sub-sets.

The Ruapehu (New Zealand) 1996 deposit, one of the largest sample datasets ever collected on a single tephra deposit^15^, is used here to quantify the effects of sampling on the calculation of TGSDs. In particular, various parts of the deposits are selectively removed from the entire dataset (subset I) to assess the influence of deposit exposure on derived TGSDs. The decrease in the number of sites (subsets II, III) mostly results in a decrease of the sorting of the distribution, while the exclusion of proximal sites (subsets IV, V, VI) results in a decrease in median grain-size and sorting. Given that most of the mass of the deposit is confined within 30 km from the vent, there is very little difference between the distributions computed for the entire dataset (I) and the subset VII, calculated based only on proximal sites (Fig. 6b,c). Md_ϕ_ increases (and, therefore, the median grain-size decreases) from about −1.5 (entire deposit) to 3.5 (when calculated for the distal deposit only), while σ_ϕ_ decreases from about 2.5 to 1 (Fig. 6b). The variability among the distributions is marked by a striking linear relation between fitted μ and σ of the lognormal distribution (Fig. 6b). These parameters show a large variability of several ϕ units with a trend which is linear (Fig. 6c). The distribution properties described based on the best fitting Rosin-Rammler distributions vary both in shape and scale (i.e., *l* and *x*_0_), even if within a much more limited range, with *l* varying from 0.7 to 1.5 and *x*_0_ from 0.0002 to 0.0033 m (Fig. 6d). Both simple and cumulative power-law fittings show a better stability of the fitting parameters (*D* and *λ*; Fig. 6e,f) calculated over the entire size distribution, with respect to fittings done excluding large (bomb) and/or small (fine ash) sizes.

**Figure 7:**
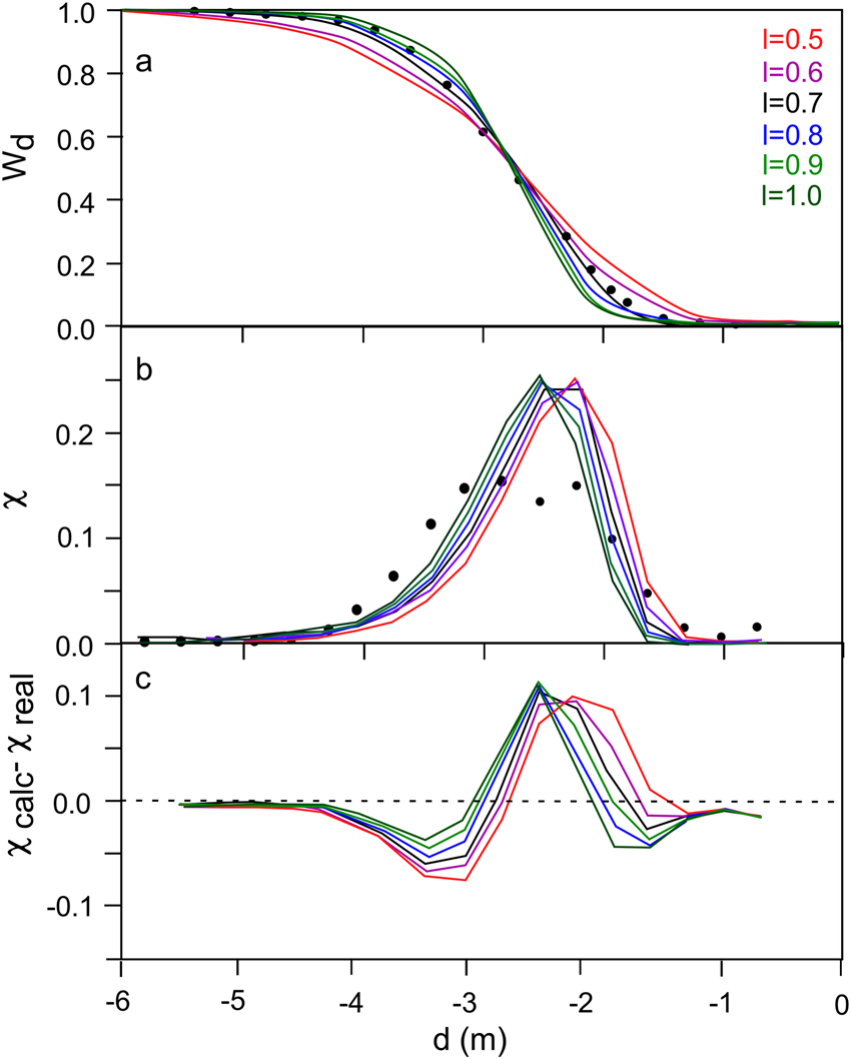
Results of variation of *l* parameter in the Rosin-Rammler distribution fitting of the 1996 Ruapehu TGSD. (**a**) Real distribution (black dots) and distribution fitted after fixing the *l* parameter to 0.5, 0.6, 0.7, 0.8, 0.9, and 1 (colored curves); (**b**) weight-fraction based (χ) distributions for the same fittings as in (**a**); (**c**) residuals (difference of calculated weight fraction χ_calc_ and the empirical weight fraction (χ_real_) of the distribution fitting, according to the same distribution as in (**a**). *l* values are indicated by different colors according to legend of (**a**). W_d_ is the weight fraction of particles of diameter smaller or equal to d.

Further exploration regarding the uncertainty associated with the fit of TGSDs with a Rosin-Rammler distribution was carried out by comparing the empirical TGSD of the 1996 Ruapehu dataset (black circles in Fig. 7) with the Rosin-Rammler distribution best fits obtained after fixing the *l* parameter at 0.5, 0.6, 0.7, 0.8, 0.9, and 1.0 (i.e., regularly spaced number between the main variability intervals defined in Fig. 3a) (Fig. 7). In other words, the uncertainty in the parameter *l* is artificially reduced by assuming that it varies within the range covered by the majority of known TGSDs. The results demonstrate that changing *l* in such a small interval has relatively minor effect on the quality of fitting, which accurately describes the tails of the distribution even though in all cases fails in reproducing the slight bimodality of the TGSD (Fig. 7). TGSDs can thus be satisfactorily reconstructed only based on the median grain-size of the studied deposit within a given range of the shape parameter *l* provided by literature data.

#### Sensitivity analysis based on synthetic deposits

In order to test the reliability of TGSDs obtained with different fitting techniques based on different sampling strategies, we simulate the dispersal of a synthetic deposit of known TGSD. For simplicity, the TGSD reproduces that of the 2450 BP Plinian eruption of Pululagua volcano (Ecuador), which was combined with four different scenarios based on variable ESPs (e.g., variable column heights and wind speeds at the tropopause).

**Figure 8:**
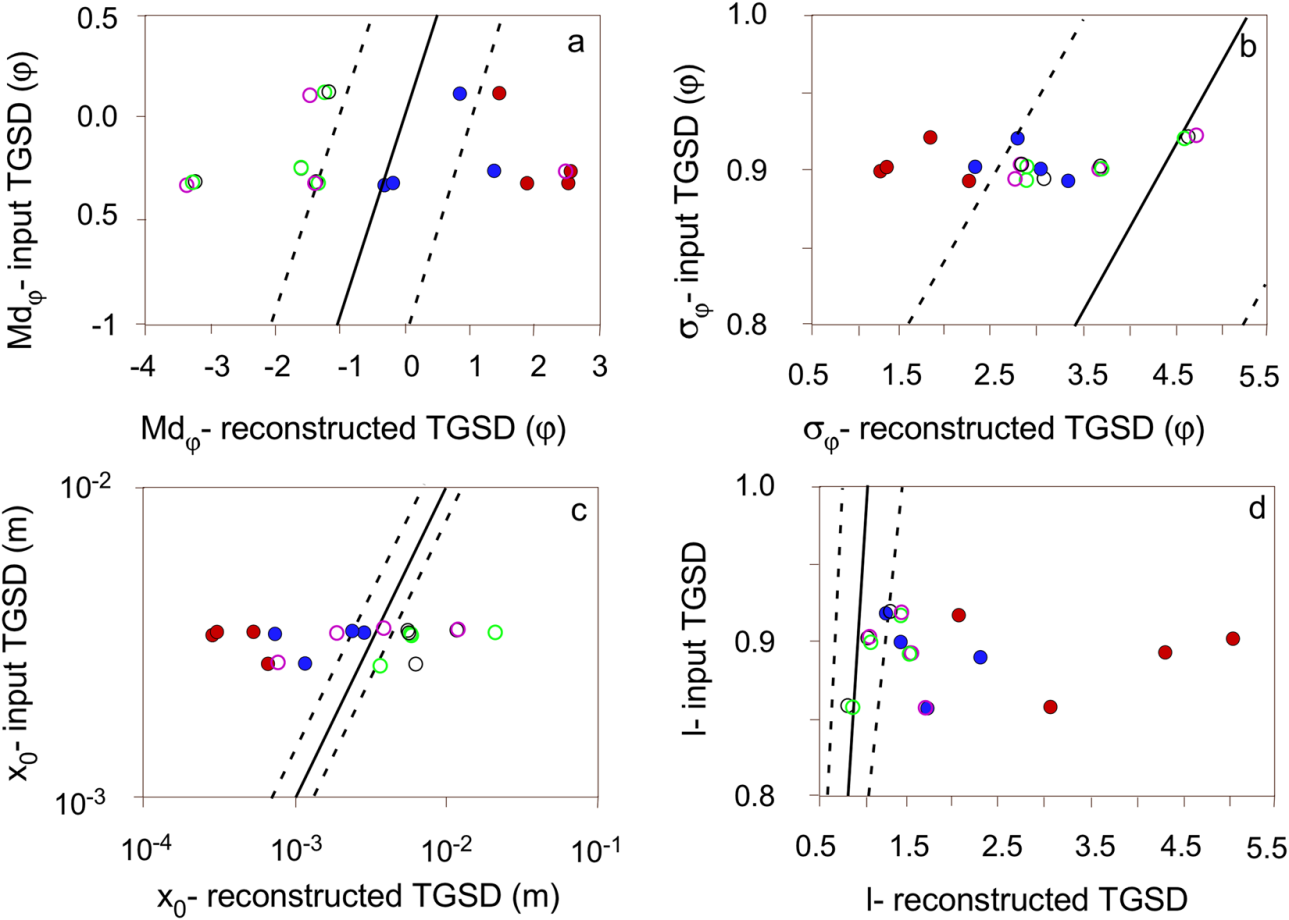
Variation of the reconstructed TGSD (**a**) Md_ϕ_ and (**b**) σ_ϕ_ vs. the input TGSDs (no fitting); (**c**) *x*_*0*_ and (**d**) *l* of the Rosin-Rammler fitting vs. the input TSGD. The black lines mark 1:1 ratio between the reconstructed and the deposit parameters, dotted lines enclose reconstructed TGSDs parameters with values within 30% (almost 1 ϕ) of the corresponding deposit ones. Symbols: red full circles = CW sampling; blue full circles = WS sampling; pink open circles = DW sampling with 10 points; black open circles = DW sampling with 20 points; green open circles = DC sampling.

The most significant result of our sensitivity analysis based on synthetic deposits is that the reconstructed TGSDs never fully matches the input distribution (Fig. 8). Md_ϕ_ is reproduced within ±30% ϕ error by 30% of the reconstructed TGSDs, and σ_ϕ_ is reproduced within ±30% of relative error by 70% of the reconstructed TGSDs (Fig. 8a,b). It is important also to note that while Md_ϕ_ could be either over- or underestimated with respect to the input one, σϕ can only be improved (i.e., it decreases). The widely spaced geometry (WS) is the best sampling strategy at reproducing the Md_ϕ_ and the σ_ϕ_ of the distribution, followed by points downwind, along the dispersal axis (DW) and points along the dispersal and a crosswind axis (DC) geometries. The points along two crosswind axes sampling geometry (CW) never gives satisfactory results, with discrepancies of distribution parameters always being larger than 30% (Fig. 8a,b).

The fitting parameters of the Rosin-Rammler distributions differ from the deposit TGSD of up to 30% in only 30% of the cases (Fig. 8c, d). The parameter *x*_0_ of the reconstructed TGSD, which could be empirically linked to the median size (Eq. 1), could either under- or overestimate that of the deposit, while the shape parameter *l* is equal or larger than the one corresponding to the input TGSD. The sampling strategies which best reproduce the input distribution parameters are the DW, DC, and WS. The parameters of the distributions derived from the CW sampling strategy display the largest discrepancy with respect to the input distribution parameters.

Finally, the TGSD obtained from 10 or 20 points distributed based on the sampling strategy DW have very similar Md_ϕ_, and slightly different σ_ϕ_ (Fig. 8). We also note that in one case (WS sampling strategy for the 30 km-high plume and 30 m/s wind intensity) the reconstructed distribution is slightly bimodal even though the original distribution has a unimodal shape.

## Discussion

Our systematic analysis of the largest available dataset of reconstructed TGSDs of tephra deposits shows a large variability of both shape and length scales. The associated Md_ϕ_ values range from −4 to 5 ϕ and σ_ϕ_ from 0.8 to 3.4 ϕ, with no apparent relationship with ESPs (Table 1).

### Effect of sampling strategy on TGSD reconstruction

Given that particle transport in volcanic clouds is mostly controlled by their terminal velocity, atmospheric profile and plume dynamics, the size of the sedimented particles negatively correlates with the sedimentation distance (i.e., large particles fall closer to the vent with respect to small ones)^13,23,24^. For this reason, the GSD at individual locations within tephra deposits varies with distance from the vent and along the crosswind direction, and at any point cannot be considered as representative of the TGSD. Moreover, due to a combination of advection-diffusion processes, the GSD at any location in the deposit is expected to be approximately lognormal around the median size corresponding to the typical terminal velocity for that distance from the vent. Such a lognormal character can, however, be affected by various size-selective sedimentation processes that can significantly change the shape of the distribution (e.g., particle aggregation, gravitational instabilities^25^). As a result, multiple GSDs need to be integrated in order to estimate the TGSD. The representativeness of the TGSD, and, therefore, the potential of the reconstructed TGSD to well reflect eruptive dynamics, depends on the number and distribution of the sampling locations considered.

The extreme sensitivity of the Md_ϕ_ and σ_ϕ_ of the reconstructed TGSD to the distribution and number of sampled sites could introduce significant bias in the results^15,26^ (Figs 6a and 8). In our sensitivity study of the TGSD reconstruction associated with the 1996 Ruapehu eruption tephra blanket, we demonstrate that both parameters are affected not only by the areal extent of the sampling, but also by the number of samples used for the computation. In the case study considered here, reducing the number of samples but not the sampled area (subsets I, II and III) results in variations of up of 0.8 ϕ in Md_ϕ_ but has a more limited effect on σ_ϕ_ (up to 0.3 ϕ). Reducing the areal extent has no effect when the majority of the deposit mass (subset VII) is included in the sampled area, but results in a significant decrease in both Md_ϕ_ (up to 3 ϕ) and σ_ϕ_ (up to 1 ϕ) when a large fraction of the deposit mass is excluded (subsets IV, V, and VI).

**Figure 9:**
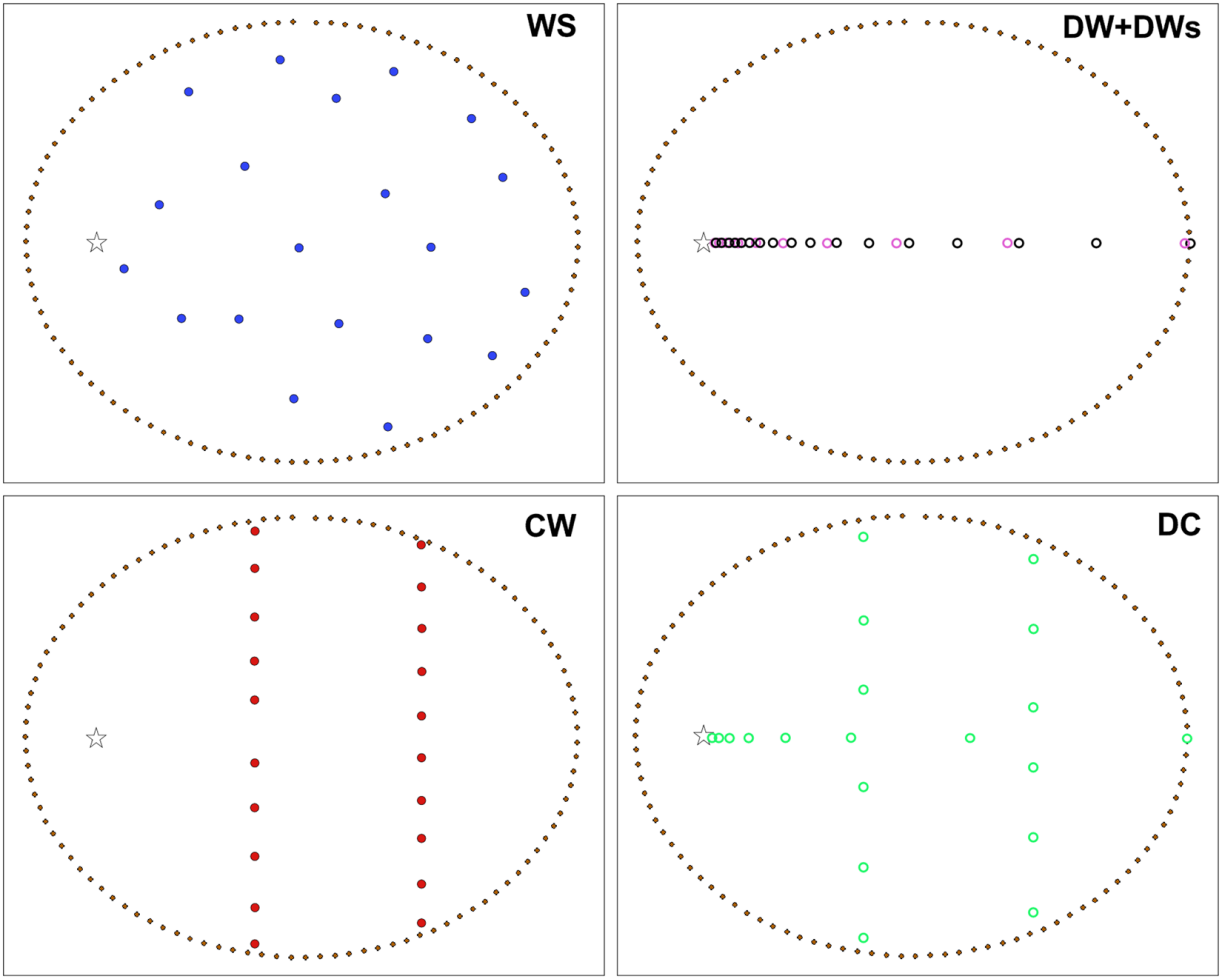
Sampling strategies over the dispersal of the deposit simulated for an eruption with column height of 10 km and maximum wind speed of 10 m/s. Stars indicate the position of the volcanic vent. Colored dots indicate the sampling points. The labels on top right correspond to the sampling strategy acronym (section 5.4). Black dots enclose the deposit areal dispersal.

The study on the synthetic deposits generated for variable eruptive conditions show that none of the tested sampling strategies (DW, CW, WS, DC; Fig. 9) could exactly reproduce the TGSD based on integration of GSDs from 20 locations, a number which is loosely representative of most published studies^12^. Of the tested sampling strategies used on synthetic deposits calculated for four eruption cases, the WS (randomly widely spaced sampling points) and the DW (points chosen along the dispersal axis) are best reproducing the input TGSD in terms of both Md_ϕ_ and σ_ϕ_ (Fig. 8).

Finally, we note that in all the case studies, and given that an incomplete sampling likely results into a smaller range of detected grain-sizes considered, the reconstructed TGSD is always better or equally sorted with respect to the TGSD of the synthetic deposit (or the most extensive sampling of the 1996 Ruapehu tephra). This result suggests that good sorting is not a criterion for representativeness of the reconstructed TGSD.

Our results also suggest that the lack of any systematic pattern linking reconstructed TGSDs to ESPs (e.g., plume height or mass eruption rate; Fig. 2e) could be mostly due to the extreme diversity in deposit exposure, and, therefore, in sampling strategies used to derive TGSDs. It is, therefore, advisable not to attempt TGSD reconstruction in cases where a significant mass fraction of the deposit is either not preserved or accessible or if the sampling sites are not widely dispersed. In this case, the resulting TGSD could be misleading and, certainly, poorly representative of the size distribution of particles at the vent.

### Shape of the distributions

Four main types of distributions, generally used to describe the result of fragmentation processes, can satisfactorily fit the empirical dataset analyzed in this paper: a lognormal distribution, commonly used in sedimentology, the Rosin-Rammler distribution, and both simple and cumulative power-law distributions. Among these, the Rosin-Rammler distribution appears to be the most successful (i.e., giving consistently larger r^2^) in reproducing the distributions of the dataset (Fig. 3), and the simple and cumulative power-law distributions are the most stable even in the case of incomplete sampling. However, a very large uncertainty is introduced when converting weight-based distributions, which are primarily determined by sieving techniques, to number-based distributions, such as the power-law distributions. The source of uncertainty derives from: i) the weighing error, which introduces a larger (i.e., several orders of magnitude) bias for small particles with respect to large ones; ii) the assumption that all particles have the same volume (necessary because of the lack of information on the particle distribution within a size (ϕ) class) (Eq. 6); and iii) the lack of information regarding of density variation with size of the particles.

These results suggest that, when a distribution model is necessary either for statistical or modeling studies, TGSDs are best approximated by a Rosin-Rammler distribution. Because of its ability in describing the tails of the distribution, the Rosin Rammler fitting is also particularly useful when it is necessary to estimate the fraction of fine or very fine particles (e.g., for health hazard assessment). However, because partial or inefficient sampling of the tephra deposit could affect both shape and scale of the reconstructed TGSD, a critical assessment on the representativeness of the distribution should be carried out based on the characteristic of the empirical dataset.

An exploration of the uncertainty associated with the Rosin Rammler distribution has been carried out by fitting the 1996 Ruapehu TGSD after fixing *x*_0_ and varying the *l* parameter between 0.5, and 1.0 (the main variability interval defined in Fig. 3a). The results demonstrate that changing *l* in such small interval has relatively minor effect of the quality of fitting, which accurately describes the tail of the distribution but fails in reproducing the slight bimodality of the TGSD (Fig. 7). This implies that even if *l* is not known, the tails of the distribution can be satisfactorily described by modeling the TGSD by a Rosin-Rammler distribution after empirical estimation of the deposit median grain-size (or *x*_*o*_) assuming that *l* lies in the range of most of published TGSDs (0.5–1; Fig. 3).

## Conclusive Remarks

The reconstruction of TGSDs is crucial to many aspects of explosive volcanism (e.g., magma fragmentation, explosive potential, tephra dispersal, volcanic impact), and, therefore, our general understanding is based on a critical interpretation of the associated empirical and theoretical distributions used to fit field observations. Theoretical fitting helps to better describe TGSDs with the potential of providing more information on the distribution characteristics (e.g., distribution tails) and minimize uncertainties associated with sampling. Given the lack of studies linking specific distribution shapes (i.e., lognormal, Rosin-Rammler, Gamma, etc.) with explosive magmatic fragmentation styles (i.e., Plinian, Strombolian, Vulcanian, etc.), TGSDs can be traditionally modelled using empirical distributions based on rock fragmentation studies. Among them, the lognormal, Rosin-Rammler, and power-law distributions adequately describe known TGSDs. Power-law distributions, despite their good fitting potential and stability (e.g., Figs 4 to 6), are affected by large uncertainties associated with the conversion of particle mass to numbers. Amongst all tested strategies used to fit particle distributions, the Rosin-Rammler shows the best compromise between fitting capacity (e.g., highest Pearson correlation coefficient) and stability with respect to sampling bias. We have also shown that TGSDs can be satisfactorily reconstructed only based on *x*_*o*_ (which is related to the median grain-size) within a given range of the shape parameter *l* provided by literature data; in particular, the Rosin-Rammler distribution cannot well describe bimodality but can best reproduce the TGSD tails even when the parameter *l* is not well constrained.

The choice of empirical and theoretical parameters to best describe TGSDs depends on the final objective. As an example, empirical parameters (i.e., Md_ϕ_ and σ_ϕ_) are the most objective and can be used for a first order characterization of the distribution, while theoretical parameters (i.e, l, x_0_, μ and σ) are more stable and can be better used to estimate specific parameters of the distribution (e.g., estimate of the percentiles of ash or block-sized pyroclasts based on the distribution tails). There are no obvious relations between published TGSD parameters (e.g., Md_ϕ_ and σ_ϕ_) and ESPs. We note that many of them (Table 1) were calculated after a limited number of samples with limited deposit exposure and 2 different reconstruction methodologies were uses, such as the Voronoi tessellation method and the weighted average, and thus might only partially reproduce the real TGSD of the corresponding deposits and not be fully comparable. This fact, combined with the complexity of magmatic fragmentation processes, could explain the very limited potential in unraveling functional relationships of known TGSDs with ESPs and conduit processes in general.

Numerical simulations and a sensitivity study carried out on a synthetic tephra deposit have confirmed that the reconstruction of the TGSD is extremely sensitive to sampling strategy. First, the reconstruction of TGSD should cover the whole deposit (i.e., WS strategy in Fig. 8), while sampling along the dispersal axis and crosswind sections do not necessarily provide good results. Second, TGSDs computed over widely distributed sampling sites appear to provide reliable median sizes but not necessarily representative sorting. Finally, TGSD is best reconstructed when most of the erupted mass is accounted for (i.e., considering sampling locations with the highest mass loads). This implies that proximal sampling is more critical to the representativity of the final TGSD than distal sampling. However, given the grain-size decrease with distance from the source, proximal sampling alone might not be able to describe the fine tail of the distribution.

## Methods and Study Dataset

### Study Dataset

The amount of observations of tephra deposits has considerably increased during the last decade and grain-size distributions from eruptions of different styles (Hawaiian to Strombolian, Vulcanian to Subplinian and Plinian) are now better characterized^11,12^. Data from 50 eruptions (available from published data; Table 1) encompassing a wide range of eruptive styles, intensities, and magma compositions have been analyzed and compared to find common properties and empirical correlations. These eruptions were fed by magmas with compositions ranging from basalt to rhyolite and tracyhte and crystal content ranging from 0.5 to 40 vol.%; they were characterized by variable intensity and style, ranging from Hawaiian to Vulcanian, Subplinian and Plinian. Some distributions are associated with co-pyroclastic density currents (co-PDCs) deposits (Table 1).

As already mentioned, the representativeness of each available TGSD depends not only on the validity of the numerical method of integration of outcrop data, but also on the sampling geometry (number and distribution of sampling points). The latter is often limited by several factors, including geographical constraints (as in the case of volcanic islands and large urban or vegetated areas), accuracy of stratigraphic reconstruction/correlation, sampling issues (e.g., in the case of very thick or very fine layers), erosional processes, and deposit contamination by co-PDC fallout, ash re-suspension or late ash explosive phases. Even in the case of a tephra deposited from a single plume, TGSD can result from several fragmentation processes and their interaction, including co-PDC coexisting with a main sustained plume and non-steady plume dynamics. Both these processes could eventually form deposits with bimodal size distributions, such as the well-documented case of the 1980 Mount St. Helens eruption^25,27^. Given the difficulty to assess the influence of deposit exposure and of eruption dynamics on the reconstructed TGSD for each specific eruption, all available TGSDs were indistinctly included in our analysis to explore first order characteristics.

For uniformity, all the TGSDs of the dataset were analyzed as 1 ϕ interval distribution, recalculating frequencies for the distribution originally published with half ϕ size intervals.

### Statistical models

TGSDs have been traditionally described as weight percent of ϕ classes (where ϕ corresponds to –log_2_ of the particle diameter), in analogy with classical sedimentology methods. Measured outcrop and deposit distributions are primarily described by two main empirical parameters in ϕ units, median (Md_ϕ_) and sorting (σ_ϕ_), which were defined to describe single location grain-size distributions (GSDs)^17,19^.

Size distributions of fragmented particles can be conveniently fitted in several ways^28^. The two key parameters, which fully constrain any statistical distribution used to describe TGSDs, are generically called the shape of the distribution and the distribution length scale. The first parameter describes the actual form of the distribution, and changes based on the distribution model used, whereas the second one generally depends either to the mean or median particle size^29^. Defined mathematical expression of these parameters correspond to any theoretical grainsize distribution, such as the l, x_0_ parameters of the Rosin-Rammler distribution and the μ and σ parameters of the log-normal distribution.

Several empirical or theoretical distributions have been proposed to fit both natural size distributions and experimental fragmentation data. They either describe the variation of particle number with particle size (if the fragmentation event produced a small number of particles which could be individually counted), or particle mass with particle size (when the particles produced are too many and their empirical distribution is constrained by sieving and weighing). These different approaches lead to a substantial gap between experimental studies, mostly based on particle number distribution, to empirical studies on fragmentation in natural systems, mostly based on the analysis of mass distributions.

Among the particle number distributions, the most used by the volcanology community is the power-law (including the fractal distribution proposed for magmatic fragmentation^1,12,30^); whereas the Mott and the Poisson distributions, proposed for ballistic studies^31^, are traditionally employed for rock fragmentation studies.

A power-law fitting probability density function has the form:

4 $${{\rm{n}}}_{d}=k{d}^{-\lambda }$$

where *n*_*d*_ is the number of particles of diameter *d*, and the parameter *λ* is the slope of the distribution, and *k* is a scaling factor which depends on the total number of particles. The cumulative power-law distribution has the form:

5 $$N(n > d)={k}_{1}{d}^{-D}$$

where *N* (*n* > *d*) is the number of particles of diameter larger than *d*, *D* is the slope of the distribution (also called the fractal dimension), and *k*_1_ is again a scaling factor which depends on the total number of particles. Because it is not possible to count directly the number of particles of a tephra deposit, a power-law fitting requires the calculation of the normalized number of particles in each size class given their weight derived from sieving data. The exact calculation requires shape, density, and size distribution within each ϕ class to be known and cannot be easily generalized. For this reason, the average particle diameter is calculated based on the average ϕ value between each class and its coarser neighbor assuming a generic spherical shape. The distributions are calculated based on the normalized number of particles *n*_*dm*_ in each size class that corresponds to:

6 $${n}_{dm}=\frac{{n}_{\varphi }}{{N}_{0}}=\frac{wt{ \% }_{\varphi }}{{(d/2)}^{3}\pi {\rho }_{\varphi }\cdot 100}$$

where *n*_ϕ_ is the number of particles calculated after the sieving weight distribution, *N*_*o*_ is the total number of particles erupted, *dm* is the average diameter of the ϕ size considered (usually expressed in m), and ρ_ϕ_ is the average density of particles in each ϕ class.

We note that, even disregarding the uncertainty in the size distribution within each ϕ class, a high uncertainty is associated with the conversion of the weight in number of particles. In detail, the uncertainty in weight propagates exponentially and scales of a factor of 3 with ϕ. Consequently, an uncertainty of 0.1% in the weight corresponds to an uncertainty in number of particles of <1 for −10 ϕ but 10^12.5^ for particles with 4 ϕ diameter.

The dependency of pyroclasts vesicularity and density on their size has already been shown^32^. Clast density typically varies from two end members, the lowest corresponding to the average vesicularity of lapilli size particles and the largest corresponding to the glass or powder density (or DRE, dense rock equivalent). The transition from these two values is, with good approximation, linear to sigmoidal in ϕ but the threshold ϕ sizes are not constant and change within each eruption depending on the original bubble size distribution and number density^32,33^. Calculating particle numbers based on a (uncalibrated) fixed density-ϕ trend would introduce artificial kinks in the distribution. For this reason, especially when comparing distribution of clasts with very different textures. The assumption of constant density is more accurate unless specific studies are available for the pyroclasts of each TGSD dataset.

Among the particle mass distributions, the most used is the lognormal, usually proposed for sedimentology studies^22^, the Rosin-Rammler /Weibull distributions (recently proposed for TGSDs^12^), and the Schuhmann distribution, largely used in metallurgical and industrial applications^34,35^. In particular, the Rosin-Rammler was first introduced by^36^ to describe particle size analysis of rock comminution processes. It was subsequently formalized by^37^ in an alternative mathematical form known as the Weibull distribution. This results in two mathematical distribution which are equivalent but fitted by distinct parameters which can be compared using conversion factors^22^.

All these distributions were developed either based on empirical or theoretical studies of fragmentation of rigid materials and have different descriptive potentials in terms of fragmentation dynamics, energy and key distribution characteristics. Some of these distributions (such as the lognormal and the Rosin-Rammler^22^) are considered as valid over the entire particle range displayed in the dataset, because they describe not only the central part but also the tails of the distributions. They are fitted based on two distinct parameters corresponding to the shape of the distribution, and the distribution length scale: the first parameter describes the actual form of the distribution and changes based on the distribution model used, whereas the second one is generally equal either to the mean or median particle size or to a definite percentile^29^. Other distributions (i.e., the power-law) are valid only in the central size range but do not describe the tails of the distributions and are still fitted by two characteristic parameters. The limits of power-law distributions are defined by the smaller and larger sizes than a given threshold value, i.e., the *fragmentation length scales*^29^. The upper-length threshold (i.e., the largest particle size) is primarily controlled by the initial magma body length scale (i.e., the length scales of the volume of magma involved). The lower-length threshold (i.e., the smallest size at which the distribution is valid) is instead controlled by the textural, physical, elastic properties of the magma, and total energy, and the stress rate associated with the fragmentation event. In the case of magmatic fragmentation, the determination of these length scales requires precise constraints of pre-eruptive conditions and magma properties, and their effect on the fragmentation dynamics, which are typically not available.

In this article we describe the four distributions which best describe the dataset. Similar analyses were also carried out for the Schumann and Mott distributions which are commonly used to model rock fragmentation^31^, but did not provide acceptable fits (Pearson correlation coefficient r^2^ are always lower than the models proposed in this paper) and, therefore, are only presented as Supplementary Material.

The probability density function of the lognormal distribution is:

7 $${w}_{\varphi }=\frac{1}{\sigma \sqrt{2\pi }}{e}^{-\frac{{({d}_{\varphi }-\mu )}^{2}}{2{\sigma }^{2}}}$$

where *w*_ϕ_ is the weight fraction of the material in each ϕ class, *d*_ϕ_ is the particle diameter in ϕ, μ is the median grain-size, and σ is the standard deviation of the distribution.

The cumulative probability density function of the Rosin-Rammler distribution can be described as:

8 $${w}_{d}={e}^{-{(\frac{d}{{x}_{0}})}^{l}}$$

where *w*_*d*_ is the weight fraction of particles of diameter smaller or equal to a given diameter (*d*) in m. The two fitting parameters provide information on the distribution shape (*l*), which is a pure number, and on the length scale (*x*_0_), which is expressed in the same length units as the particle diameter (here expressed in m). In literature, *x*_0_ has been empirically linked with various percentiles of the original distribution^22^.

### Empirical study on the effect of incomplete sampling

The effect of the number and distribution of sampled sites used to compute the TGSD can be evaluated by calculating the TGSD of the same deposit based on a variable number of site GSDs. This test was performed on the 1996 Ruapehu eruption tephra deposit (New Zealand), which represents the most extensive dataset used for the compilation of a TGSD^15,38^. We considered the distributions calculated using I) the entire dataset, consisting of 105 sampling sites distributed over an area of about 10^4^ km^2^ and located up to 160 km from the vent; II) 50% of the original sites, maintaining a similar distribution arrangement; III) 10% of the sites, maintaining a similar distribution arrangement; IV) sites at distances from the vent exceeding 5 km; V) sites at distances from the vent exceeding 30 km; VI) sites at 50 km or more from the vent; and VII) sites at distances smaller than 30 km from the vent. The extent of sampling for the different combinations is shown in Fig. 6a. The deposit is marked by a rapid mass load decay with distance from the source. The tephra deposited within 5 km, 30 km, and 50 km from the vent, calculated after the isomass distribution shown in Fig. 6a based on the strategy of^39^, accounts for 42, 52, and 70% of the total deposit, respectively.

### Numerical simulations

In order to test the performance of different sampling techniques, we simulate the dispersal of a synthetic deposit of known TGSD. For simplicity, the TGSD reproduces that of the 2450 BP Plinian eruption of Pululagua volcano (Ecuador), having Md_ϕ_ = 0.24 and σ_ϕ_ = 1.91^18^. The distribution is unimodal and, due to the limited amount of fine ash, aggregation processes affecting deposition patterns by mobilizing of fine particles into larger aggregates can be considered negligible.

We initially combine four different scenarios based on variable ESPs. These include two column heights (10 km and 25 km above the vent) and two wind speeds at the tropopause (10 m/s and 30 m/s, with a standard vertical profile and constantly blowing W—E; Fig. S1). The dispersal of the erupted mass (4.5 × 10^11^ kg^18^) on a hypothetical flat topography from an ideal vent at 750 m above sea level is produced by using the numerical model TEPHRA2^40,41^. TEPHRA2 relies on an analytical solution of the advection–diffusion equation, which accounts for various regimes of sedimentation based on the terminal velocity of particles and size-dependent atmospheric diffusion. Regardless of its simplicity, TEPHRA2 has been successfully applied to simulate the particle deposition of both strong plumes (e.g., Tarawera volcano, New Zealand^40^; Cotopaxi volcano, Ecuador^42^) and weak plumes (Ruapehu volcano, New Zealand^40^; Etna volcano, Italy^43^). Before setting up the sampling geometries, we initially made four runs with TEPHRA2 to check in which area most of the erupted mass (>95 wt%) was emplaced. In order to do this, we integrated the deposit grain-size computed at the nodes of a 1-km-spaced grid, 600 × 600 km (600 × 1000 km in the case of a column of 25 km with a wind speed of 30 m/s) for a total of 3.6 × 10^5^ points (Fig. S2).

Once the tephra accumulation on the ground and the grain-size distribution at each grid point was obtained, we applied the Voronoi tessellation technique to reconstruct the TGSD^15^. As zero lines (required by the tessellation technique) we used the isolines of mass loading >0.001 g/m^2^ obtained for each run. We first verified that with the complete grids the reconstructed TGSDs were virtually identical to the input distribution (i.e., marked by very similar distribution shapes and parameters), and as a next step we designed four different geometries of 20 points on the ground corresponding to variable sampling strategies (Fig. 8). This number is representative of the average sites used to reconstruct TGSD in literature (ref.^12^ and dataset analyzed in this work). In particular, we chose a distance of 5 km from the vent as the most proximal site and the zero line as the farthest point, given that deposits are rarely sampled in very proximal areas due to poor accessibility and/or preservation. The geometries include: (i) a distribution of 20 widely spaced points covering the entire deposit area (WS); (ii) a downwind distribution (DW) along the dispersal axis with exponentially increasing distance between points; (iii) two N—S crosswind distributions (CW), 10 points each at 1/3 and 2/3 of the total distance; (iv) a combined geometry of 8 points along the downwind axis with exponentially increasing distance coupled with two perpendicular, N-S distributions of six points each (DC). The four geometries were used with all the combinations of column heights and wind speeds, for a total of 16 cases. The DW geometry was also tested with only 10 points (DWs) to assess the reliability of smaller datasets (Fig. 9).

For each eruption case and sampling strategy, the TGSD obtained by integrating the points using the Voronoi tessellation method (Fig. S3) was compared with the input TGSD (i.e., the TGSD obtained by integrating the grain-size of the deposit in all nodes of the grid constrained by the 0.001 g/m^2^ contour).

## Supplementary information

Supplementary Dataset 1

Supplementary Dataset 2
