# Author Correction: Reliability of Total Grain-Size Distribution of Tephra Deposits

**DOI:** 10.1038/s41598-020-69881-4

**Published:** 2020-08-07

**Authors:** L. Pioli, C. Bonadonna, M. Pistolesi

**Affiliations:** 1 Dipartimento di Scienze Chimiche e Geologiche, Università di Cagliari, Cagliari, Italy; 2 Département des Sciences de la Terre, Université de Genève, Genève, Switzerland; 3 Dipartimento di Scienze della Terra, Università di Pisa, Pisa, Italy

Correction to: *Scientific Reports*https://doi.org/10.1038/s41598-019-46125-8, published online 10 July 2019

This Article contains errors.

Firstly, there is an error in Equation 1.

$${x}_{0}=\frac{1}{3} \alpha$$

should read:

$${x}_{0}=K \alpha$$

As a result of the error in Equation 1, the following accompanying definition is also incorrect:

“with r^2^ = 0.9996”

should read:

“$K$ equals 1.4 with r^2^ = 0.99 when calculated based on selected TGSDs of Table 1 (i.e. TGSDs which could be fitted by a Rosin-Rammler distribution with a fitting correlation coefficient ≥ 0.99).”

In the Results section:

“In literature, the length scale parameter ${x}_{0}$ (Eq. 8) has been empirically linked with various percentiles of the original distribution^22^.”

should read:

“In literature, the length scale parameter ${x}_{0}$ (Eq. 8) has been linked with various percentiles of the original distribution^22^, however, here we estimate it based on the best fit of empirical data.”

“For the reasons above, the fitting improves when considering only particles coarser than fine ash (≤ 5 φ; Fig. 4b) or particles with size comprised from lapilli (< − 6 φ) to coarse ash (< − 1 φ) (Fig. 4c).”

should read:

“For the reasons above, the fitting improves when considering only particles coarser than fine ash (≤ 4 φ; Fig. 4b) or particles with size comprised from lapilli (> − 6 φ) to coarse ash (≤ 4 φ) (Fig. 4c).”

The following sentence in the Results should also be omitted:

“TGSDs can thus be satisfactorily reconstructed only based on the median grain-size of the studied deposit within a given range of the shape parameter *l* provided by literature data.”

In the Discussion section:

“This implies that even if *l* is not known, the tails of the distribution can be satisfactorily described by modeling the TGSD by a Rosin-Rammler distribution after empirical estimation of the deposit median grain-size (or ${x}_{0}$) assuming that *l* lies in the range of most of published TGSDs (0.5–1; Fig. 3).”

should read:

“In case the TGSD is not well fitted by a Rosin-Rammler distribution (r^2^ < 0.99), the tails of the distribution can be satisfactorily described after estimation of ${x}_{0}$ based on the deposit median grain-size (Eq. 1) assuming that *l* lies in the range of most of published TGSDs (0.5–1; Fig. 3).”

In the Conclusive Remarks:

“Amongst all tested strategies used to fit particle distributions, the Rosin-Rammler shows the best compromise between fitting capacity (e.g., highest Pearson correlation coefficient) and stability with respect to sampling bias.”

should read:

“Amongst all tested strategies used to fit particle distributions, the Rosin-Rammler shows the best compromise between fitting capacity (e.g., highest correlation coefficient) and stability with respect to sampling bias.”

In the Methods section:

“Similar analyses were also carried out for the Schumann and Mott distributions which are commonly used to model rock fragmentation^31^, but did not provide acceptable fits (Pearson correlation coefficient r^2^ are always lower than the models proposed in this paper) and, therefore, are only presented as Supplementary Material.”

should read:

“Similar analyses were also carried out for the Schumann and Mott distributions which are commonly used to model rock fragmentation^31^, but did not provide acceptable fits (correlation coefficient r^2^ are always lower than the models proposed in this paper) and, therefore, are only presented as Supplementary Material.”

In Figure Legends 4 and 6:

“Pearson correlation coefficient”

should read:

“correlation coefficient”

In the Figure 5 Legend:

“Variability of D versus Pearson correlation coefficient r^2^ obtained from the power-law fitting of cumulative number of particles all the TGSDs considered (Eq. 5, Table 1); (a) entire distribution; (b) particles coarser than fine ash (< 0.64 mm); (c) lapilli (64–2 mm) to coarse ash (2–0.063 mm) particles.”

should read:

“Variability of D versus correlation coefficient r^2^ obtained from the power-law fitting of cumulative number of particles all the TGSDs considered (Eq. 5, Table 1); a) entire distribution; b) particles coarser than fine ash (> 0.063 mm); c) lapilli (64–2 mm) to coarse ash (2–0.063 mm) particles.”

In Supplementary Information Table S1, the value under column “${x}_{0}$ (m)” for row “Hekla 1991” should read 0.00515.

Furthermore, the axes in Figure 3 were incorrectly switched, and the data point mentioned above in Supplementary Table S1 is incorrect in Figure 3. The correct Figure 3 is reproduced below as Fig. 1.

**Figure 1:**
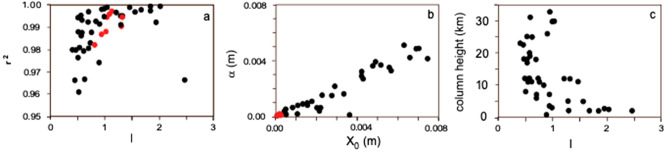
(**a**) Pearson correlation coefficient r^2^ of the Rosin-Rammler distribution fitting vs. *l* of the studied distributions. (**b**) Variability of the parameter *x*_0_ of the Rosin-Rammler distribution fitting vs. the empirical median diameter (α) of the studied distributions. The dashed line indicates the best-fit linear correlation between the two parameters. (**c**) Variability of the parameter *l* vs. column height of the associated eruptions. Symbols as in Fig. 2.

Lastly, Figure 7 is incorrect as a result of the error in Equation 1. The correct Figure 7 is reproduced below as Fig. 2.

**Figure 2:**
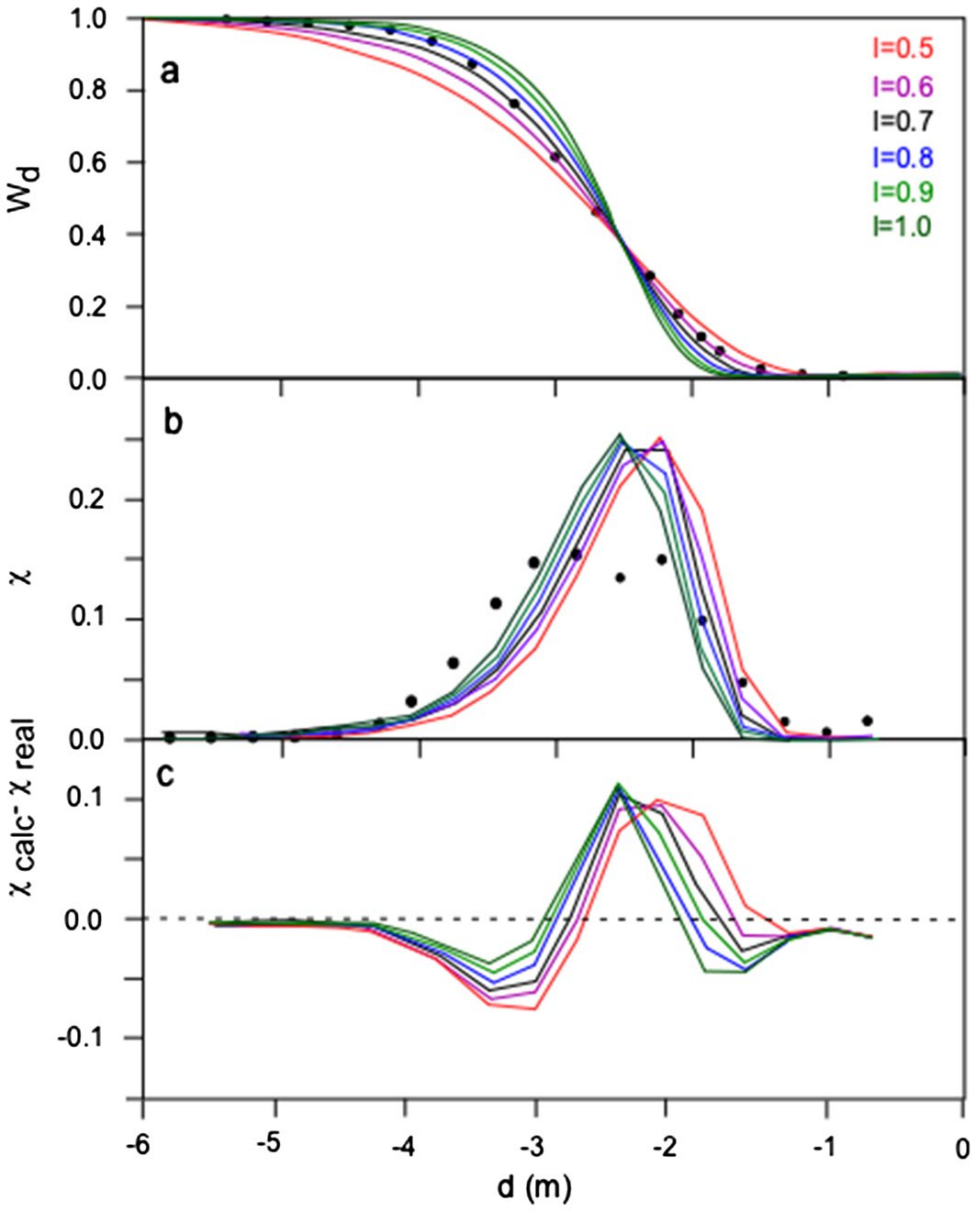
Results of variation of *l* parameter in the Rosin-Rammler distribution fitting of the 1996 Ruapehu TGSD. (**a**) Real distribution (black dots) and distribution fitted after fixing the *l* parameter to 0.5, 0.6, 0.7, 0.8, 0.9, and 1 (colored curves); (**b**) weight-fraction based (χ) distributions for the same fittings as in (**a**); (**c**) residuals (difference of calculated weight fraction χ_calc_ and the empirical weight fraction (χ_real_) of the distribution fitting, according to the same distribution as in (**a**). *l* values are indicated by different colors according to legend of (**a**). W_d_ is the weight fraction of particles of diameter smaller or equal to d.

