# Supplementary material for: Reliability of Total Grain-Size Distribution of Tephra Deposits
Source: Sci Rep. 2019 Jul 10;9:10006. doi: 10.1038/s41598-019-46125-8 (PMC6620348; doi:10.1038/s41598-019-46125-8)
Supplement: Supplementary file 1 — Supplementary Dataset 1 [file 41598_2019_46125_MOESM1_ESM.docx]

Reliability of Total Grainsize Distribution of Tephra Deposits’

L. Pioli, C. Bonadonna and M. Pistolesi.

Supplementary information

**STATISTICAL TABLES**

**Notation**

*d*_φ_= diameter of the particle, φ units

*d*= diameter of the particle, linear units (m in this work)

*D*= shape parameter of the cumulative power law distribution (fractal dimension)

*l*= distribution shape parameter of the Rosin-Rammler distribution

Md_φ_ = median grain-size of the empirical distribution, (φ units)

n=number of particles

*n_d_*= number of particles of diameter d

*n_dm_* = normalized number of particles in size class dm

*n*_φ_ = number of particles of diameter φ

*N (n>d)* = number of particles with diameter larger than d

*w*_φ_= weight fraction of material in each φ class

*w_d_*= weight fraction of particles of diameter smaller or equal to d

*x_0_*= length scale parameter of the Rosin-Rammler distribution

*α*= median grain-size of the empirical distribution (linear units)

χ= weight fraction of particle in each size class

*λ*= shape parameter of the power-law distribution

*μ*=median of the lognormal distribution (φ units)

*σ*= standard deviation of the lognormal distribution (φ units)

σ_φ_= sorting of the empirical distribution, (φ units)

| **Eruption** | **l** | **X_0_ (m)** | **r^2^** | **RMSE** |
| --- | --- | --- | --- | --- |
| Etna 19-24/07/2001 | 1.945 | 0.00042 | 0.992 | 0.042 |
| Etna 27/10/2002 | 0.978 | 0.00153 | 0.995 | 0.030 |
| Etna 24/11/2006 | 2.469 | 0.00049 | 0.966 | 0.062 |
| Etna 4-5/09/2007 | 1.286 | 0.00229 | 0.991 | 0.043 |
| Etna 12-13/01/2011 | 0.940 | 0.00692 | 0.998 | 0.017 |
| Etna 18-19/05/2016 | 1.673 | 0.00107 | 0.998 | 0.013 |
| Etna 21/05/2016 | 2.026 | 0.00082 | 0.999 | 0.011 |
| Izu Oshima 1986 | 1.312 | 0.02357 | 0.995 | 0.019 |
| Fuego 1974 | 1.206 | 0.00134 | 0.997 | 0.205 |
| Heimaey 1973 | 1.838 | 0.00489 | 0.998 | 0.023 |
| Hekla 1845 | 0.567 | 0.00276 | 0.995 | 0.027 |
| Hekla 1991 | 0.545 | 0.04101 | 0.988 | 0.009 |
| Hekla 2000 | 0.488 | 0.00216 | 0.944 | 0.090 |
| Hekla 1104 | 0.452 | 0.00103 | 0.966 | 0.070 |
| Hekla 1300 D | 0.514 | 0.00804 | 0.977 | 0.061 |
| Hekla 1693 | 0.465 | 0.00204 | 0.998 | 0.055 |
| Hekla 1766 | 0.518 | 0.00219 | 0.988 | 0.040 |
| Tecolote | 1.459 | 0.00421 | 0.999 | 0.014 |
| Kilauea Iki, 1959 | 0.891 | 1.86800 | 0.974 | 0.010 |
| Eyjafjallajokull 4-8/05/2010 | 0.504 | 0.00046 | 0.994 | 0.026 |
| St. Vincent 1979 | 0.751 | 0.00026 | 0.988 | 0.041 |
| Chaiten 06/05/2011 | 0.687 | 0.00033 | 0.993 | 0.036 |
| Al Madinah 1256 | 1.840 | 0.00631 | 1.000 | 0.004 |
| Ruapehu 1996 | 0.695 | 0.00335 | 0.997 | 0.023 |
| Mt Spurr Aug 1992 | 0.532 | 0.00048 | 0.981 | 0.056 |
| Mt Spurr Sept 1992 | 0.730 | 0.00017 | 0.983 | 0.057 |
| Soufriere Hills 31/03/1997 | 1.299 | 0.00008 | 0.991 | 0.045 |
| Soufriere Hills 12/09/1997 | 1.129 | 0.00011 | 0.997 | 0.023 |
| Soufriere Hills 15/09/1997 | 1.052 | 0.00008 | 0.995 | 0.032 |
| Soufriere Hills 21/09/1997 | 1.310 | 0.00003 | 0.995 | 0.034 |
| Soufriere Hills 26/09/1997 | 0.611 | 0.00026 | 0.980 | 0.059 |
| Soufriere Hills 28/09/1997 | 0.810 | 0.00008 | 0.982 | 0.059 |
| Soufriere Hills 01/10/1997 | 1.012 | 0.00009 | 0.988 | 0.048 |
| Soufriere Hills 02/10/1997 | 0.936 | 0.00010 | 0.987 | 0.050 |
| Soufriere Hills 10/10/1997 | 1.080 | 0.00012 | 0.996 | 0.028 |
| Soufriere Hills 18/07/2005 | 0.834 | 0.00006 | 0.993 | 0.036 |
| Soufriere Hills 27/07/2005 | 0.697 | 0.00006 | 0.981 | 0.979 |
| Mt. St. Helens 18/05/1980 | 0.572 | 0.00012 | 0.988 | 0.042 |
| Cordón Caulle 2011 Unit I | 0.563 | 0.00744 | 0.986 | 0.034 |
| Askja 1875 phase C | 0.627 | 0.00363 | 0.967 | 0.059 |
| Askja 1875 phase D | 0.402 | 0.01771 | 0.980 | 0.050 |
| Vesuvius 1906 L2 | 1.280 | 0.00423 | 0.995 | 0.022 |
| Vesuvius 1906 L3 | 1.557 | 0.00290 | 0.997 | 0.019 |
| Vesuvius 1906 ash | 0.940 | 0.00003 | 0.998 | 0.018 |
| Baia | 0.520 | 0.00477 | 0.961 | 0.067 |
| Pululagua 2450 BP | 0.900 | 0.00158 | 0.992 | 0.042 |
| El Chichon 1982 | 0.576 | 0.00014 | 0.993 | 0.032 |
| Cotopaxi layer 3 | 1.040 | 0.00705 | 0.999 | 0.011 |
| Cotopaxi layer 5 | 0.955 | 0.00658 | 0.999 | 0.011 |
| Rungwe pumice | 0.992 | 0.00430 | 0.998 | 0.016 |

Table 1. Main parameters of the Rosin-Rammler distribution fitting of TGSDs. For symbols, see article text. r^2^= Pearson correlation coefficient, RMSE= Root Mean Square Error.

| **Eruption** | **σ (phi)** | **μ (phi)** | **r^2^** | **RMSE** |
| --- | --- | --- | --- | --- |
| Etna 19-24/07/2001 | 0.916 | 2.175 | 0.991 | 0.010 |
| Etna 27/10/2002 | 1.809 | 0.522 | 0.984 | 0.009 |
| Etna 24/11/2006 | 0.633 | 1.714 | 0.967 | 0.022 |
| Etna 4-5/09/2007 | 1.328 | -0.031 | 0.983 | 0.011 |
| Etna 12-13/01/2011 | 1.782 | -1.879 | 0.922 | 0.021 |
| Etna 18-19/05/2016 | 1.030 | 0.816 | 0.952 | 0.034 |
| Etna 21/05/2016 | 1.007 | 1.114 | 0.973 | 0.034 |
| Izu Oshima 1986 | 1.106 | -3.690 | 0.937 | 0.020 |
| Fuego 1974 | 1.364 | 0.568 | 0.988 | 0.009 |
| Heimaey 1973 | 0.844 | -1.537 | 0.983 | 0.014 |
| Hekla 1845 | 1.560 | -1.680 | 0.567 | 0.039 |
| Hekla 1991 | 1.396 | -2.785 | 0.567 | 0.039 |
| Hekla 2000 | 3.665 | 1.500 | 0.408 | 0.053 |
| Hekla 1104 | 1.613 | 4.382 | 0.148 | 0.046 |
| Hekla 1300-D | 1.579 | 3.585 | 0.143 | 0.048 |
| Hekla 1693 | - | - | - | - |
| Hekla 1766 | - | - | - | - |
| Tecolote | 1.032 | -1.359 | 0.921 | 0.021 |
| Kilauea Iki, 1959 | 0.489 | -8.804 | 0.984 | 0.019 |
| Eyjafjallajokull 4-8/05/2010 | 3.487 | 2.617 | 0.872 | 0.017 |
| St. Vincent 1979 | 2.374 | 3.508 | 0.923 | 0.018 |
| Chaiten 06/05/2011 | 2.456 | 3.215 | 0.990 | 0.005 |
| Al Madinah 1256 | 0.886 | -1.940 | 0.985 | 0.013 |
| Ruapehu 1996 | 2.474 | -0.311 | 0.979 | 0.003 |
| Mt Spurr Aug 1992 | 3.022 | 3.296 | 0.695 | 0.031 |
| Mt Spurr Sept 1992 | 1.845 | 4.632 | 0.846 | 0.025 |
| Soufriere Hills 31/03/1997 | 1.357 | 4.442 | 0.698 | 0.055 |
| Soufriere Hills 12/09/1997 | 1.605 | 4.148 | 0.974 | 0.013 |
| Soufriere Hills 15/09/1997 | 1.776 | 4.855 | 0.880 | 0.028 |
| Soufriere Hills 21/09/1997 | 1.290 | 5.254 | 0.990 | 0.009 |
| Soufriere Hills 26/09/1997 | 2.823 | 3.983 | 0.800 | 0.026 |
| Soufriere Hills 28/09/1997 | 1.792 | 5.558 | 0.886 | 0.023 |
| Soufriere Hills 01/10/1997 | 1.718 | 4.754 | 0.967 | 0.014 |
| Soufriere Hills 02/10/1997 | 1.727 | 4.905 | 0.936 | 0.018 |
| Soufriere Hills 10/10/1997 | 1.692 | 4.152 | 0.933 | 0.021 |
| Soufriere Hills 18/07/2005 | 1.875 | 5.321 | 0.951 | 0.014 |
| Soufriere Hills 27/07/2005 | 2.115 | 5.667 | 0.844 | 0.014 |
| Mt. St. Helens 18/05/1980 | 3.050 | 5.051 | 0.904 | 0.016 |
| Cordón Caulle 2011 Unit I | 2.197 | -2.034 | 0.917 | 0.016 |
| Askja 1875 phase C | 2.064 | 3.496 | 0.624 | 0.017 |
| Askja 1875 phase D | 4.383 | -1.865 | 0.844 | 0.016 |
| Vesuvius 1906 L2 | 1.268 | -1.049 | 0.983 | 0.011 |
| Vesuvius 1906 L3 | 1.102 | -0.585 | 0.995 | 0.007 |
| Vesuvius 1906 ash | 1.701 | 6.071 | 0.922 | 0.020 |
| Baia | 1.301 | -2.357 | 0.622 | 0.062 |
| Pululagua 2450 BP | 1.821 | 0.824 | 0.952 | 0.015 |
| El Chichon 1982 | 3.109 | 4.448 | 0.881 | 0.018 |
| Cotopaxi layer 3 | 1.621 | -2.000 | 0.951 | 0.017 |
| Cotopaxi layer 5 | 1.803 | -2.000 | 0.955 | 0.015 |
| Rungwe pumice | 1.740 | -1.020 | 0.993 | 0.006 |

Table 2. Main parameters of lognormal (in phi) distribution fitting of TGSDs. r^2^ = Pearson correlation coefficient, RMSE= Root Mean Square Error, For details, see article. Where no data is shown, the fitting had no reliability (i.e., r^2^ is lower than 1).

| **Eruption** | **All particles** | | **Particles coarser than fine ash** | | **Lapilli to coarse ash particles** | |
| --- | --- | --- | --- | --- | --- | --- |
|  | **λ** | **r^2^** | **λ** | **r^2^** | **λ** | **r^2^** |
| Etna 19-24/07/2001 | 3.166 | 0.912 | 3.714 | 0.962 | 3.714 | 0.962 |
| Etna 27/10/2002 | 2.854 | 0.960 | 3.078 | 0.968 | 3.078 | 0.968 |
| Etna 24/11/2006 | 2.058 | 0.797 | 2.690 | 0.885 | 2.690 | 0.885 |
| Etna 4-5/09/2007 | 2.705 | 0.930 | 2.915 | 0.939 | 2.915 | 0.939 |
| Etna 12-13/01/2011 | 2.768 | 0.957 | 2.768 | 0.957 | 2.768 | 0.957 |
| Etna 18-19/05/2016 | 2.467 | 0.894 | 2.844 | 0.926 | 2.844 | 0.926 |
| Etna 21/05/2016 | 2.949 | 0.876 | 3.328 | 0.887 | 3.328 | 0.887 |
| Izu Oshima 1986 | 2.406 | 0.985 | 2.406 | 0.985 | 2.282 | 0.989 |
| Fuego 1974 | 2.184 | 0.977 | 2.537 | 0.962 | 2.537 | 0.962 |
| Heimaey 1973 | 2.937 | 0.934 | 2.937 | 0.934 | 2.937 | 0.934 |
| Hekla 1845 | 2.536 | 0.970 | 3.188 | 0.988 | 3.187 | 0.988 |
| Hekla 1991 | 2.574 | 0.981 | 3.111 | 0.981 | 3.111 | 0.981 |
| Hekla 2000 | 2.647 | 0.950 | 3.486 | 0.958 | 3.486 | 0.958 |
| Hekla 1104 | 2.807 | 0.984 | 3.291 | 0.989 | 3.291 | 0.989 |
| Hekla 1300-D | 2.899 | 0.971 | 3.609 | 0.970 | 3.609 | 0.970 |
| Hekla 1693 | 2.655 | 0.983 | 3.167 | 0.993 | 3.167 | 0.993 |
| Hekla 1766 | 2.642 | 0.967 | 3.266 | 0.988 | 3.266 | 0.988 |
| Tecolote | 2.823 | 0.913 | 2.823 | 0.913 | 2.823 | 0.913 |
| Kilauea Iki, 1959 | 1.792 | 0.962 | 1.792 | 0.962 | 1.526 | 0.920 |
| Eyjafjallajokull 4-8/05/2010 | 2.788 | 0.994 | 3.109 | 0.996 | 3.109 | 0.996 |
| St. Vincent 1979 | 2.625 | 0.963 | 3.213 | 0.998 | 3.213 | 0.998 |
| Chaiten 06/05/2011 | 2.968 | 0.979 | 3.658 | 0.996 | 3.658 | 0.996 |
| Al Madinah 1256 | 2.030 | 0.956 | 2.030 | 0.956 | 2.030 | 0.956 |
| Ruapehu 1996 | 2.422 | 0.960 | 3.260 | 0.988 | 3.089 | 0.985 |
| Mt Spurr Aug 1992 | 2.901 | 0.979 | 3.476 | 0.988 | 3.476 | 0.988 |
| Mt Spurr Sept 1992 | 3.009 | 0.982 | 3.459 | 0.994 | 3.399 | 0.992 |
| Soufriere Hills 31/03/1997 | 2.848 | 0.737 | 6.546 | 0.947 | 6.546 | 0.947 |
| Soufriere Hills 12/09/1997 | 3.195 | 0.868 | 5.767 | 0.959 | 5.767 | 0.959 |
| Soufriere Hills 15/09/1997 | 3.191 | 0.922 | 5.026 | 0.991 | 5.026 | 0.991 |
| Soufriere Hills 21/09/1997 | 3.353 | 0.954 | 4.269 | 0.991 | 4.269 | 0.991 |
| Soufriere Hills 26/09/1997 | 2.942 | 0.953 | 3.622 | 0.985 | 3.622 | 0.985 |
| Soufriere Hills 28/09/1997 | 3.486 | 0.979 | 4.034 | 0.977 | 4.034 | 0.977 |
| Soufriere Hills 01/10/1997 | 3.271 | 0.917 | 4.858 | 0.978 | 4.858 | 0.978 |
| Soufriere Hills 02/10/1997 | 3.041 | 0.953 | 3.840 | 0.991 | 3.840 | 0.991 |
| Soufriere Hills 10/10/1997 | 3.051 | 0.910 | 4.610 | 0.991 | 4.610 | 0.991 |
| Soufriere Hills 18/07/2005 | 3.181 | 0.983 | 3.865 | 0.986 | 3.865 | 0.986 |
| Soufriere Hills 27/07/2005 | 3.271 | 0.980 | 3.922 | 0.961 | 3.922 | 0.961 |
| Mt. St. Helens 18/05/1980 | 3.077 | 0.988 | 3.573 | 0.995 | 3.573 | 0.995 |
| Cordón Caulle 2011 Unit I | 2.716 | 0.993 | 2.547 | 0.974 | 2.547 | 0.974 |
| Askja 1875 phase C | 3.413 | 0.998 | 3.525 | 1.000 | 3.502 | 1.000 |
| Askja 1875 phase D | 2.921 | 0.996 | 3.022 | 0.998 | 2.926 | 0.997 |
| Vesuvius 1906 L2 | 2.530 | 0.960 | 2.530 | 0.960 | 2.530 | 0.960 |
| Vesuvius 1906 L3 | 2.611 | 0.911 | 2.611 | 0.911 | 2.611 | 0.911 |
| Vesuvius 1906 ash | 3.064 | 0.981 | - | - | - | - |
| Baia | 2.761 | 0.989 | 2.775 | 0.988 | 2.775 | 0.988 |
| Pululagua 2450 BP | 2.741 | 0.946 | 3.567 | 0.976 | 3.224 | 0.975 |
| El Chichon 1982 | 3.118 | 0.979 | 3.744 | 0.995 | 3.744 | 0.995 |
| Cotopaxi layer 3 | 2.485 | 0.970 | 2.741 | 0.915 | 2.741 | 0.915 |
| Cotopaxi layer 5 | 2.342 | 0.974 | 2.745 | 0.952 | 2.745 | 0.952 |
| Rungwe pumice | 2.854 | 0.952 | 2.937 | 0.943 | 2.937 | 0.943 |

Table 3. Parameters of power law distribution fitting of TGSDs. r^2^ = Pearson correlation coefficient. For details, see article. Where no data is shown, the fitting was no possible (no large particles in the distribution).

| **Eruption** | **All particles** | | **Particles coarser than fine ash** | | **Lapilli to coarse ash particles** | |
| --- | --- | --- | --- | --- | --- | --- |
|  | **D** | **r^2^** | **D** | **r^2^** | **D** | **r^2^** |
| Etna 19-24/07/2001 | 3.368 | 0.942 | 3.823 | 0.972 | 3.823 | 0.972 |
| Etna 27/10/2002 | 2.641 | 0.976 | 2.838 | 0.983 | 2.838 | 0.983 |
| Etna 24/11/2006 | 2.385 | 0.872 | 2.898 | 0.910 | 2.898 | 0.910 |
| Etna 4-5/09/2007 | 2.808 | 0.940 | 3.031 | 0.951 | 3.031 | 0.951 |
| Etna 12-13/01/2011 | 2.841 | 0.962 | 2.841 | 0.962 | 2.841 | 0.962 |
| Etna 18-19/05/2016 | 2.633 | 0.917 | 2.971 | 0.937 | 2.971 | 0.937 |
| Etna 21/05/2016 | 3.084 | 0.891 | 3.482 | 0.905 | 3.482 | 0.905 |
| Izu Oshima 1986 | 2.437 | 0.984 | 2.437 | 0.984 | 2.304 | 0.990 |
| Fuego 1974 | 2.232 | 0.978 | 2.613 | 0.965 | 2.613 | 0.965 |
| Heimaey 1973 | 3.024 | 0.939 | 3.024 | 0.939 | 3.024 | 0.939 |
| Hekla 1845 | 2.786 | 0.992 | 2.771 | 0.989 | 2.771 | 0.989 |
| Hekla 1991 | 2.583 | 0.973 | 3.211 | 0.988 | 3.211 | 0.988 |
| Hekla 2000 | 2.693 | 0.951 | 3.502 | 0.958 | 3.502 | 0.958 |
| Hekla 1104 | 2.835 | 0.985 | 3.304 | 0.989 | 3.304 | 0.989 |
| Hekla 1300-D | 2.928 | 0.971 | 3.627 | 0.970 | 3.627 | 0.970 |
| Hekla 1693 | 2.688 | 0.984 | 3.181 | 0.993 | 3.181 | 0.993 |
| Hekla 1766 | 2.679 | 0.978 | 3.286 | 0.987 | 3.286 | 0.987 |
| Tecolote | 2.934 | 0.923 | 2.934 | 0.923 | 2.934 | 0.923 |
| Kilauea Iki, 1959 | 1.749 | 0.945 | 1.749 | 0.945 | 1.628 | 0.936 |
| Eyjafjallajokull 4-8/05/2010 | 2.953 | 0.998 | 3.146 | 0.996 | 3.146 | 0.996 |
| St. Vincent 1979 | 2.752 | 0.979 | 3.248 | 0.998 | 3.248 | 0.998 |
| Chaiten 06/05/2011 | 3.198 | 0.986 | 3.675 | 0.996 | 3.675 | 0.996 |
| Al Madinah 1256 | 2.161 | 0.959 | 2.161 | 0.959 | 2.161 | 0.959 |
| Ruapehu 1996 | 2.494 | 0.969 | 3.282 | 0.989 | 3.124 | 0.986 |
| Mt Spurr Aug 1992 | 3.141 | 0.988 | 3.500 | 0.988 | 3.500 | 0.988 |
| Mt Spurr Sept 1992 | 3.244 | 0.994 | 3.471 | 0.994 | 3.598 | 0.994 |
| Soufriere Hills 31/03/1997 | 3.289 | 0.851 | 6.560 | 0.948 | 6.560 | 0.948 |
| Soufriere Hills 12/09/1997 | 3.352 | 0.893 | 5.808 | 0.961 | 5.808 | 0.961 |
| Soufriere Hills 15/09/1997 | 3.328 | 0.940 | 5.050 | 0.991 | 5.050 | 0.991 |
| Soufriere Hills 21/09/1997 | 3.457 | 0.966 | 4.286 | 0.991 | 4.286 | 0.991 |
| Soufriere Hills 26/09/1997 | 3.069 | 0.971 | 3.648 | 0.985 | 3.648 | 0.985 |
| Soufriere Hills 28/09/1997 | 3.522 | 0.980 | 4.064 | 0.978 | 4.064 | 0.978 |
| Soufriere Hills 01/10/1997 | 3.412 | 0.938 | 4.886 | 0.979 | 4.886 | 0.979 |
| Soufriere Hills 02/10/1997 | 3.161 | 0.969 | 3.862 | 0.992 | 3.862 | 0.992 |
| Soufriere Hills 10/10/1997 | 3.226 | 0.940 | 4.646 | 0.992 | 4.646 | 0.992 |
| Soufriere Hills 18/07/2005 | 3.205 | 0.984 | 3.883 | 0.986 | 3.883 | 0.986 |
| Soufriere Hills 27/07/2005 | 3.286 | 0.981 | 3.973 | 0.968 | 3.973 | 0.968 |
| Mt. St. Helens 18/05/1980 | 3.290 | 0.995 | 3.599 | 0.996 | 3.599 | 0.996 |
| Cordón Caulle 2011 Unit I | 2.762 | 0.999 | 2.606 | 0.979 | 2.606 | 0.979 |
| Askja 1875 phase C | 3.428 | 0.998 | 3.534 | 1.000 | 3.505 | 1.000 |
| Askja 1875 phase D | 2.940 | 0.996 | 3.038 | 0.998 | 2.940 | 0.998 |
| Vesuvius 1906 L2 | 2.595 | 0.961 | 2.595 | 0.961 | 2.595 | 0.961 |
| Vesuvius 1906 L3 | 2.726 | 0.923 | 2.726 | 0.923 | 2.726 | 0.923 |
| Vesuvius 1906 ash | 3.099 | 0.981 | - | - | - | - |
| Baia | 2.787 | 0.992 | 2.771 | 0.989 | 2.771 | 0.989 |
| Pululagua 2450 BP | 2.220 | 0.970 | 2.827 | 0.973 | 2.827 | 0.973 |
| El Chichon 1982 | 3.386 | 0.993 | 3.765 | 0.995 | 3.765 | 0.995 |
| Cotopaxi layer 3 | 2.534 | 0.965 | 2.822 | 0.922 | 2.822 | 0.922 |
| Cotopaxi layer 5 | 2.393 | 0.997 | 2.806 | 0.955 | 2.806 | 0.955 |
| Rungwe pumice | 2.903 | 0.953 | 3.008 | 0.948 | 3.008 | 0.948 |

Table 4. Parameters of cumulative power law distribution fitting of TGSDs. r^2^ = Pearson correlation coefficient. For details, see article. Where no data is shown, the fitting was no possible (no large particles in the distribution).

Supplemental models fitting results

The cumulative form of the Mott distribution (Mott, 1943) has the form:

$w_{d}=e^{-({\frac{d}{x_{0}})}^{o.5}}$

where *w_d_* is the weight fraction of particles of diameter greater or equal to a given diameter (*d*) in m. x_0_ is the length scale of the distribution. This distribution is based on two-dimensional statistics.

Mott, N.F. and Linfoot, E.H., 1943, A theory of fragmentation. British Ministry of Supply Report, AC 3348.

| **Eruption** | **X_0_ (m)** | **r^2^** | **RMSE** |
| --- | --- | --- | --- |
| Etna 19-24/07/2001 | 0.00074 | 0.940 | 0.0119 |
| Etna 27/10/2002 | 0.00212 | 0.971 | 0.0802 |
| Etna 24/11/2006 | 0.00078 | 0.936 | 0.1169 |
| Etna 4-5/09/2007 | 0.00365 | 0.956 | 0.0907 |
| Etna 12-13/01/2011 | 0.00904 | 0.972 | 0.0747 |
| Etna 18-19/05/2016 | 0.00177 | 0.917 | 0.1283 |
| Etna 21/05/2016 | 0.00138 | 0.909 | 0.1376 |
| Izu Oshima 1986 | 0.03478 | 0.950 | 0.0952 |
| Fuego 1974 | 0.00196 | 0.962 | 0.0922 |
| Heimaey 1973 | 0.00782 | 0.937 | 0.1180 |
| Hekla 1845 | 0.00293 | 0.992 | 0.0361 |
| Hekla 1991 | 0.00534 | 0.986 | 0.0425 |
| Hekla 2000 | 0.00212 | 0.978 | 0.0653 |
| Hekla 1104 | 0.00094 | 0.964 | 0.0702 |
| Hekla 1300-D | 0.00082 | 0.976 | 0.0594 |
| Hekla 1693 | 0.00194 | 0.978 | 0.0553 |
| Hekla 1766 | 0.00223 | 0.987 | 0.0401 |
| Telocote | 0.00619 | 0.825 | 0.1844 |
| Kilauea Iki, 1959 | 1.20400 | 0.774 | 0.1287 |
| Eyjafjallajokull 4-8/05/2010 | 0.00046 | 0.998 | 0.0197 |
| St. Vincent 1979 | 0.00033 | 0.983 | 0.0599 |
| Chaiten 06/05/2011 | 0.00040 | 0.989 | 0.0474 |
| Al Madinah 1256 | 0.00992 | 0.928 | 0.1226 |
| Ruapehu 1996 | 0.00406 | 0.990 | 0.0452 |
| Mt Spurr Aug 1992 | 0.00051 | 0.988 | 0.0484 |
| Mt Spurr Sept 1992 | 0.00023 | 0.979 | 0.0629 |
| Soufriere Hills 31/03/1997 | 0.00012 | 0.952 | 0.1019 |
| Soufriere Hills 12/09/1997 | 0.00016 | 0.962 | 0.0916 |
| Soufriere Hills 15/09/1997 | 0.00011 | 0.964 | 0.0872 |
| Soufriere Hills 21/09/1997 | 0.00001 | 0.955 | 0.0972 |
| Soufriere Hills 26/09/1997 | 0.00031 | 0.985 | 0.0542 |
| Soufriere Hills 28/09/1997 | 0.00011 | 0.976 | 0.0691 |
| Soufriere Hills 01/10/1997 | 0.00014 | 0.966 | 0.0844 |
| Soufriere Hills 02/10/1997 | 0.00015 | 0.972 | 0.0765 |
| Soufriere Hills 10/10/1997 | 0.00017 | 0.964 | 0.0883 |
| Soufriere Hills 18/07/2005 | 0.00008 | 0.980 | 0.0631 |
| Soufriere Hills 27/07/2005 | 0.00008 | 0.984 | 0.0541 |
| Mt. St. Helens 18/05/1980 | 0.00013 | 0.991 | 0.0410 |
| Cordón Caulle 2011 Unit I | 0.00771 | 0.993 | 0.0344 |
| Askja 1875 phase C | 0.00048 | 0.978 | 0.0675 |
| Askja 1875 phase D | 0.01498 | 0.985 | 0.0481 |
| Vesuvius 1906 L2 | 0.00643 | 0.956 | 0.0963 |
| Vesuvius 1906 L3 | 0.00004 | 0.972 | 0.0724 |
| Vesuvius 1906 ash | 0.00469 | 0.947 | 0.1083 |
| Baia | 0.00484 | 0.961 | 0.0645 |
| Pululagua 2450 BP | 0.00224 | 0.975 | 0.0732 |
| El Chichon 1982 | 0.00016 | 0.994 | 0.0329 |
| Cotopaxi layer 3 | 0.00954 | 0.966 | 0.0826 |
| Cotopaxi layer 5 | 0.00878 | 0.971 | 0.0759 |
| Rungwe pumice | 0.00590 | 0.970 | 0.0787 |

Table 5. Fitting parameters of the Mott distribution. r^2^ = Pearson correlation coefficient, RMSE= Root Mean Square Error.

The cumulative form the Mott distribution solved for 3D geometry of breakage (Mott, 1943) has the form:

$w_{d}=e^{-({\frac{d}{x_{0}})}^{1/3}}$

where *w_d_* is the weight fraction of particles of diameter smaller or equal to a given diameter (*d*) in m. x_0_ is the length scale of the distribution.

Mott, N.F. and Linfoot, E.H., 1943, A theory of fragmentation. British Ministry of Supply Report, AC 3348.

| **Eruption** | **X_0_ (m)** | **r^2^** | **RMSE** |
| --- | --- | --- | --- |
| Etna 19-24/07/2001 | 0.00106 | 0.880 | 0.168 |
| Etna 27/10/2002 | 0.00297 | 0.916 | 0.135 |
| Etna 24/11/2006 | 0.00103 | 0.884 | 0.157 |
| Etna 4-5/09/2007 | 0.00524 | 0.897 | 0.151 |
| Etna 12-13/01/2011 | 0.01255 | 0.915 | 0.131 |
| Etna 18-19/05/2016 | 0.00254 | 0.829 | 0.184 |
| Etna 21/05/2016 | 0.00198 | 0.823 | 0.192 |
| Izu Oshima 1986 | 0.04875 | 0.880 | 0.147 |
| Fuego 1974 | 0.00276 | 0.906 | 0.145 |
| Heimaey 1973 | 0.01110 | 0.872 | 0.167 |
| Hekla 1845 | 0.00395 | 0.933 | 0.092 |
| Hekla 1991 | 0.00725 | 0.926 | 0.097 |
| Hekla 2000 | 0.00296 | 0.956 | 0.091 |
| Hekla 1104 | 0.00138 | 0.940 | 0.090 |
| Hekla 1300-D | 0.00118 | 0.933 | 0.100 |
| Hekla 1693 | 0.00266 | 0.950 | 0.084 |
| Hekla 1766 | 0.00311 | 0.934 | 0.093 |
| Telocote | 0.00874 | 0.666 | 0.255 |
| Kilauea Iki, 1959 | 2.60700 | 0.646 | 0.161 |
| Eyjafjallajokull 4-8/05/2010 | 0.00061 | 0.975 | 0.069 |
| St. Vincent 1979 | 0.00046 | 0.937 | 0.114 |
| Chaiten 06/05/2011 | 0.00058 | 0.950 | 0.010 |
| Al Madinah 1256 | 0.0019 | 0.808 | 0.170 |
| Ruapehu 1996 | 0.00570 | 0.947 | 0.101 |
| Mt Spurr Aug 1992 | 0.00075 | 0.964 | 0.082 |
| Mt Spurr Sept 1992 | 0.00035 | 0.945 | 0.102 |
| Soufriere Hills 31/03/1997 | 0.00016 | 0.893 | 0.153 |
| Soufriere Hills 12/09/1997 | 0.00023 | 0.904 | 0.145 |
| Soufriere Hills 15/09/1997 | 0.00015 | 0.907 | 0.140 |
| Soufriere Hills 21/09/1997 | 0.00014 | 0.899 | 0.147 |
| Soufriere Hills 26/09/1997 | 0.00043 | 0.950 | 0.010 |
| Soufriere Hills 28/09/1997 | 0.00016 | 0.933 | 0.110 |
| Soufriere Hills 01/10/1997 | 0.00019 | 0.911 | 0.137 |
| Soufriere Hills 02/10/1997 | 0.00022 | 0.923 | 0.126 |
| Soufriere Hills 10/10/1997 | 0.00023 | 0.908 | 0.142 |
| Soufriere Hills 18/07/2005 | 0.00012 | 0.935 | 0.112 |
| Soufriere Hills 27/07/2005 | 0.00011 | 0.945 | 0.101 |
| Mt. St. Helens 18/05/1980 | 0.00019 | 0.960 | 0.085 |
| Cordón Caulle 2011 Unit I | 0.00977 | 0.963 | 0.080 |
| Askja 1875 phase C | 0.00071 | 0.939 | 0.112 |
| Askja 1875 phase D | 0.02170 | 0.980 | 0.054 |
| Vesuvius 1906 L2 | 0.00905 | 0.896 | 0.148 |
| Vesuvius 1906 L3 | 0.00663 | 0.886 | 0.159 |
| Vesuvius 1906 ash | 0.00005 | 0.916 | 0.125 |
| Baia | 0.00635 | 0.891 | 0.106 |
| Pululagua 2450 BP | 0.00320 | 0.925 | 0.126 |
| El Chichon 1982 | 0.00021 | 0.961 | 0.085 |
| Cotopaxi layer 3 | 0.01327 | 0.907 | 0.137 |
| Cotopaxi layer 5 | 0.01224 | 0.914 | 0.132 |
| Rungwe pumice | 0.00824 | 0.914 | 0.134 |

Table 6. Fitting parameters of the 3D Mott distribution. r^2^ = Pearson correlation coefficient, RMSE= Root Mean Square Error.

The Gates-Gaudin-Schumann distribution (Schumann, 1941) is a power law distribution based on the mass distribution of particles.

The cumulative distribution has the form:

${M(x)}_{d}={(\frac{d}{\lambda})}^{n}$

where *M* is the mass fraction of the total sample mass *M_0_*  that is smaller to a given diameter (*d*) in m; λ is the length scale of the distribution.

Schumann R., 1941. Principles of comminution, I. Size distribution and surface calculations. AIME Tech Publ 1189:11. Mining Technology.

| **Eruption** | **λ (m)** | **n** | **R^2^** | **RMSE** |
| --- | --- | --- | --- | --- |
| Etna 19-24/07/2001 | 0.223 | 0.132 | 0.682 | 0.132 |
| Etna 27/10/2002 | 0.304 | 0.151 | 0.765 | 0.232 |
| Etna 24/11/2006 | 0.223 | 0.224 | 0.693 | 0.250 |
| Etna 4-5/09/2007 | 0.357 | 0.165 | 0.767 | 0.231 |
| Etna 12-13/01/2011 | 0.461 | 0.187 | 0.818 | 0.195 |
| Etna 18-19/05/2016 | 0.286 | 0.149 | 0.728 | 0.026 |
| Etna 21/05/2016 | 0.266 | 0.144 | 0.712 | 0.267 |
| Izu Oshima 1986 | 0.676 | 0.238 | 0.842 | 0.173 |
| Fuego 1974 | 0.296 | 0.150 | 0.742 | 0.240 |
| Heimaey 1973 | 0.435 | 0.186 | 0.761 | 0.229 |
| Hekla 1845 | 0.348 | 0.152 | 0.831 | 0.184 |
| Hekla 1991 | 0.411 | 0.165 | 0.852 | 0.169 |
| Hekla 2000 | 0.330 | 0.146 | 0.819 | 0.190 |
| Hekla 1104 | 0.275 | 0.130 | 0.812 | 0.189 |
| Hekla 1300-D | 0.255 | 0.129 | 0.981 | 0.202 |
| Hekla 1693 | 0.325 | 0.142 | 0.834 | 0.178 |
| Hekla 1766 | 0.328 | 0.146 | 0.829 | 0.183 |
| Telocote | 0.411 | 0.180 | 0.774 | 0.227 |
| Kilauea Iki, 1959 | 1.754 | 0.743 | 0.916 | 0.080 |
| Eyjafjallajokull 4-8/05/2010 | 0.205 | 0.116 | 0.779 | 0.017 |
| St. Vincent 1979 | 0.181 | 0.115 | 0.720 | 0.246 |
| Chaiten 06/05/2011 | 0.200 | 0.118 | 0.736 | 0.230 |
| Al Madinah 1256 | 0.464 | 0.193 | 0.775 | 0.766 |
| Ruapehu 1996 | 0.387 | 0.163 | 0.824 | 0.190 |
| Mt Spurr Aug 1992 | 0.226 | 0.121 | 0.779 | 0.209 |
| Mt Spurr Sept 1992 | 0.184 | 0.110 | 0.731 | 0.232 |
| Soufriere Hills 31/03/1997 | 0.130 | 0.100 | 0.639 | 0.287 |
| Soufriere Hills 12/09/1997 | 0.143 | 0.105 | 0.660 | 0.278 |
| Soufriere Hills 15/09/1997 | 0.129 | 0.099 | 0.649 | 0.278 |
| Soufriere Hills 21/09/1997 | 0.126 | 0.098 | 0.634 | 0.282 |
| Soufriere Hills 26/09/1997 | 0.183 | 0.113 | 0.736 | 0.234 |
| Soufriere Hills 28/09/1997 | 0.136 | 0.099 | 0.681 | 0.255 |
| Soufriere Hills 01/10/1997 | 0.137 | 0.102 | 0.647 | 0.274 |
| Soufriere Hills 02/10/1997 | 0.146 | 0.104 | 0.681 | 0.263 |
| Soufriere Hills 10/10/1997 | 0.145 | 0.106 | 0.665 | 0.275 |
| Soufriere Hills 18/07/2005 | 0.124 | 0.094 | 0.671 | 0.257 |
| Soufriere Hills 27/07/2005 | 0.124 | 0.093 | 0.682 | 0.247 |
| Mt. St. Helens 18/05/1980 | 0.146 | 0.996 | 0.719 | 0.232 |
| Cordón Caulle 2011 Unit I | 0.458 | 0.171 | 0.869 | 0.155 |
| Askja 1875 phase C | 0.217 | 0.122 | 0.744 | 0.234 |
| Askja 1875 phase D | 0.695 | 0.188 | 0.915 | 0.115 |
| Vesuvius 1906 L2 | 0.416 | 0.178 | 0.787 | 0.217 |
| Vesuvius 1906 L3 | 0.378 | 0.170 | 0.765 | 0.232 |
| Vesuvius 1906 ash | 0.094 | 0.084 | 0.616 | 0.274 |
| Baia | 0.396 | 0.161 | 0.850 | 0.169 |
| Pululagua 2450 BP | 0.318 | 0.152 | 0.769 | 0.222 |
| El Chichon 1982 | 0.151 | 0.102 | 0.722 | 0.232 |
| Cotopaxi layer 3 | 0.466 | 0.189 | 0.813 | 0.199 |
| Cotopaxi layer 5 | 0.458 | 0.186 | 0.808 | 0.197 |
| Rungwe pumice | 0.409 | 0.175 | 0.801 | 0.006 |

Table 6. Fitting parameters of the Gaudin Schumann distribution. R^2^ = Pearson correlation coefficient, RMSE= Root Mean Square Error.
