# Supplementary material for: Reliability of Total Grain-Size Distribution of Tephra Deposits
Source: Sci Rep. 2019 Jul 10;9:10006. doi: 10.1038/s41598-019-46125-8 (PMC6620348; doi:10.1038/s41598-019-46125-8)
Supplement: Supplementary file 2 — Supplementary Dataset 2 [file 41598_2019_46125_MOESM2_ESM.docx]

Reliability of Total Grainsize Distribution of Tephra Deposits’

L. Pioli, C. Bonadonna and M. Pistolesi.

Supplementary information

**NUMERICAL SIMULATIONS AND TGSD CALCULATIONS**

**
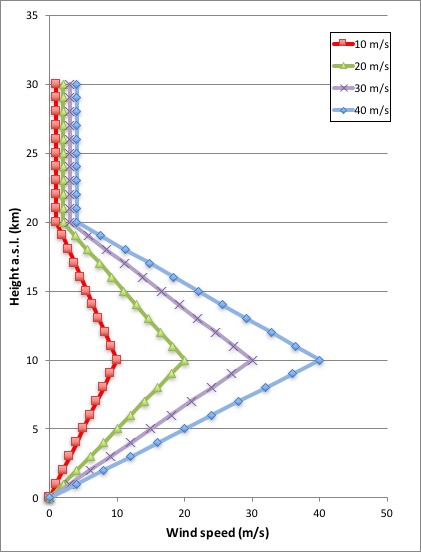
**

**Figure S1**

S1 Wind standard vertical profile and constantly blowing W-E used to derive the two wind speeds at the tropopause (10 m/s and 30 m/s) used for the TEPHRA2 runs.


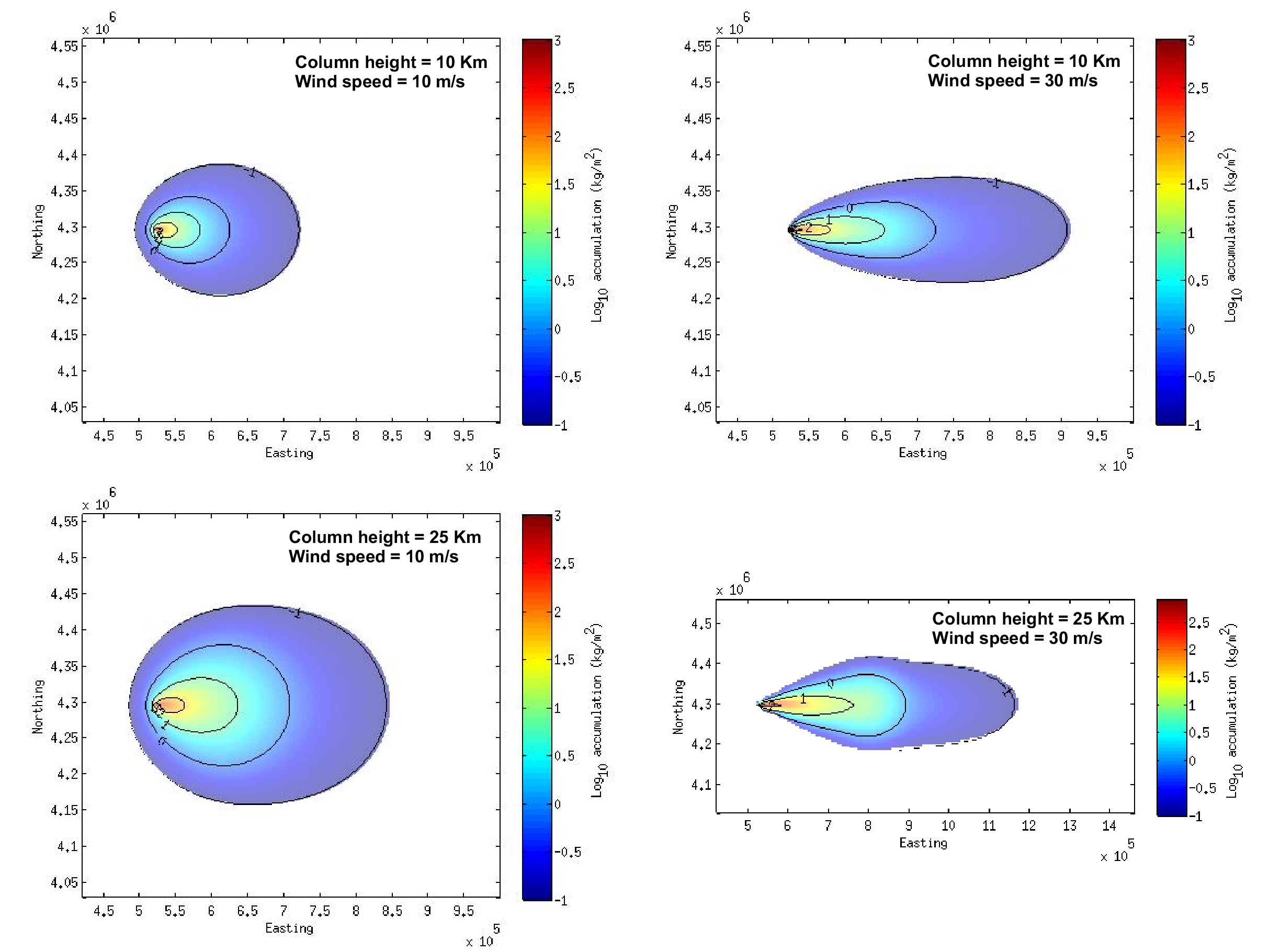


**Figure S2**

Erupted mass obtained by TEPHRA2 runs with four different combinations of wind speed and column height. For each case, >95 wt% of the initial deposit mass is emplaced on the ground when grainsize data is integrated on the deposit computed at the nodes of a 1 km spaced grid, 600x600 km (600x1000 km in the case of a column of 25 km with a wind speed of 30 m/s) for a total of 3.6x10^5^ points.


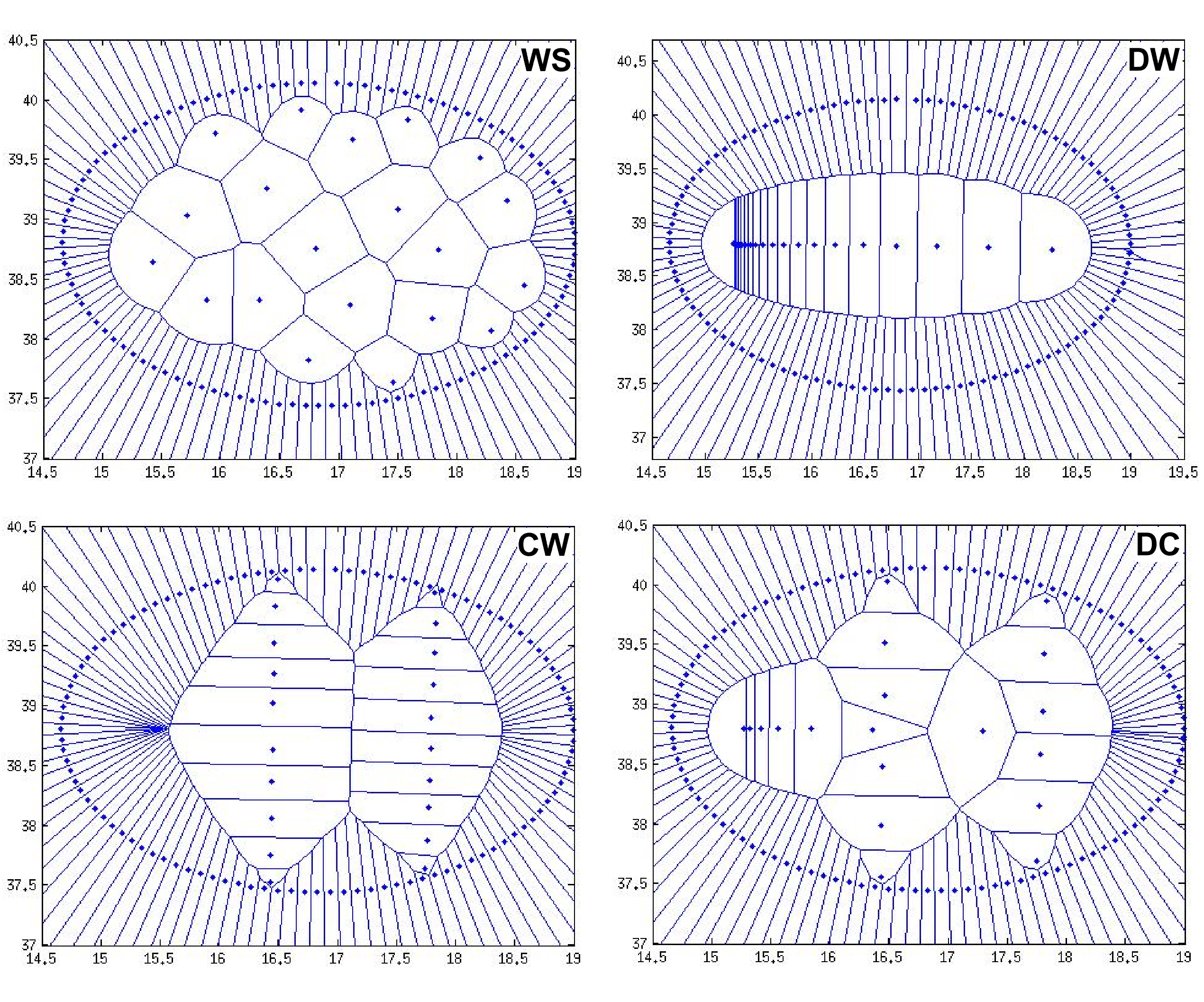


**Figure S3**

Voronoi tessellation for the four different sampling geometries of Fig. 8 in the main text. The combination of 10 km column height and 10 m/s wind speed at the tropopause is shown.
